# Nanoplasmonic SERS on fidget spinner for digital bacterial identification

**DOI:** 10.1038/s41378-025-00870-1

**Published:** 2025-03-03

**Authors:** Mamata Karmacharya, Issac Michael, Jiyun Han, Elizabeth Maria Clarissa, Oleksandra Gulenko, Sumit Kumar, Yoon-Kyoung Cho

**Affiliations:** 1 Center for Algorithmic and Robotic Synthesis (CARS), Institute for Basic Science (IBS), Ulsan, 44919 South Korea; 2 Department of Biomedical Engineering, Ulsan National Institute of Science and Technology (UNIST), Ulsan, 44919 South Korea

**Keywords:** Biosensors, Biosensors

## Abstract

Raman spectroscopy offers non-destructive and highly sensitive molecular insights into bacterial species, making it a valuable tool for detection, identification, and antibiotic susceptibility testing. However, achieving clinically relevant accuracy, quantitative data, and reproducibility remains challenging due to the dominance of bulk signals and the uncontrollable heterogeneity of analytes. In this study, we introduce an innovative diagnostic tool: a plasmonic fidget spinner (*P*-FS) incorporating a nitrocellulose membrane integrated with a metallic feature, referred to as a nanoplasmonic-enhanced matrix, designed for simultaneous bacterial filtration and detection. We developed a method to fabricate a plasmonic array patterned nitrocellulose membrane using photolithography, which is then integrated with a customized fidget spinner. Testing the *P*-FS device with various bacterial species (*E. coli* 25922, *S. aureus* 25923, *E. coli* MG1655, *Lactobacillus brevis*, and *S. mutans* 3065) demonstrated successful identification based on their unique Raman fingerprints. The bacterial interface with regions within the plasmonic array, where the electromagnetic field is most intensely concentrated—called nanoplasmonic hotspots—on the *P*-FS significantly enhances sensitivity, enabling more precise detection. SERS intensity mappings from the Raman spectrometer are transformed into digital signals using a threshold-based approach to identify and quantify bacterial distribution. Given the *P*-FS’s ability to enhance vibrational signatures and its scalable fabrication under routine conditions, we anticipate that nanoplasmonic-enhanced Raman spectroscopy—utilizing nanostructures made from metals (specifically gold and silver) deposited onto a nitrocellulose membrane to amplify Raman scattering signals—will become the preferred technology for reliable and ultrasensitive detection of various analytes, including those crucial to human health, with strong potential for transitioning from laboratory research to clinical applications.

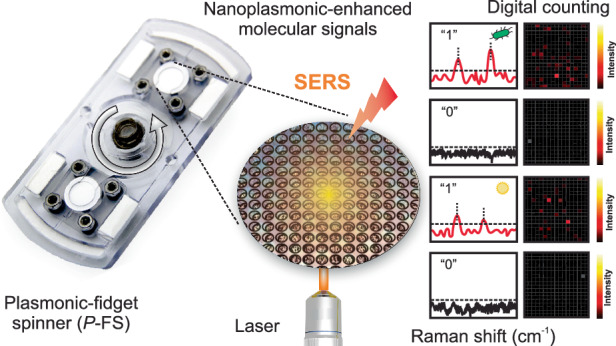

## Introduction

Pathogenic bacteria continue to be a significant public health threat, responsible for approximately 550 million infections and 5.2 million deaths annually, making them one of the leading causes of mortality worldwide^1,2^. Although these infections are often treatable, patient outcomes are heavily reliant on the timely administration of appropriate drugs, as delays can critically impact survival rates. This underscores the urgent need for accurate on-site identification to provide early warnings and protect public health. Rapid identification of infections is therefore recognized as a crucial intervention during initial care^3^.

However, current protocols for bacterial pathogen identification, such as culture in growth media, biochemical assays, and serological diagnostics, are extremely time-consuming and fail to meet the rigorous demands of the present age. These methods are limited by extended turnaround times (TAT) of 2–3 days or more and require labor-intensive procedures, which necessitate considerable expertise for practical implementation in real-world settings^4^. As a result, there is a pressing need to develop innovative techniques that can bridge the gap between basic laboratory research and clinical applications, enabling accurate and rapid bacterial identification within a defined timeframe^5^. This highlights the urgent demand to enhance analytical techniques for reliable and swift bacterial detection, ensuring both public health safety and the effectiveness of medical diagnostics.

In recent years, surface-enhanced Raman spectroscopy (SERS) has garnered significant attention in both research and clinical contexts due to its efficiency, speed, and minimally invasive nature, making it suitable for real-time applications^6,7^. SERS is a highly sensitive analytical method that utilizes substantial electromagnetic field enhancement through localized surface plasmon resonances (LSPR) to detect biomolecules at ultra-low concentrations, down to the atto-molar range^6,8,9^. This capability has been bolstered by advancements in instrumentation and data-handling techniques, making SERS a cost-effective and label-free method for chemical analysis, particularly in pathogen diagnosis^10–12^.

However, achieving clinically relevant accuracy, quantitative data, and reproducibility remains challenging due to the dominance of bulk signals and the uncontrollable heterogeneity of analytes. To address the challenges of variable signal intensity and low reproducibility at low analyte concentrations, digital Raman techniques have been proposed^13–15^. By converting analog SERS signals into digital data, single-molecule counting can be achieved, enhancing both accuracy and reliability^16^. This approach is particularly effective with metallic colloidal nanoparticles in solution, which provide a sufficient enhancement to identify and quantify individual bacterial molecules^17,18^.

Combining SERS with digital Raman methods can enables comprehensive, species-specific biochemical insights into bacterial surfaces, allowing for universal and rapid molecular identification within seconds to minutes, without the need for specific biomarkers^19,20^. This innovative integration holds significant promise for bridging the gap between experimental research and practical clinical applications, enabling dependable and swift bacterial detection and characterization^21,22^.

**Scheme 1:**
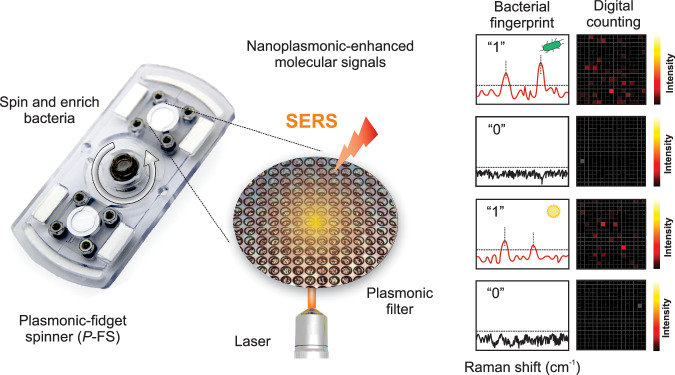
Schematic representation of the plasmonic fidget spinner (*P*-FS) used for the isolation and digital detection of bacteria through SERS mechanism. A urine sample is introduced to capture and enrich bacteria via centrifugal force from spinning the *P*-FS device. The enriched bacteria on the plasmonic filter was processed for enhanced Raman signal detection using laser-induced SERS. Unique bacterial fingerprints are captured and displayed in digital Raman spectroscopy profiles, differentiating positive (“1”) and negative (“0”) detections, with intensity represented in heatmaps

Herein, we develop an innovative SERS-based diagnostic tool: a plasmonic fidget spinner (*P*-FS) incorporating a nitrocellulose membrane integrated with a metallic feature, referred to as a nanoplasmonic-enhanced matrix, designed for simultaneous bacterial filtration and detection. Building on our previous work with diagnostic fidget spinners (Dx-FS)^23^, which enabled colorimetric detection of bacteria in urinary tract infections, the *P*-FS significantly enhances the sensitivity and specificity of detection by incorporating SERS (Scheme 1).

We developed a method to fabricate a plasmonic array patterned nitrocellulose membrane using photolithography, followed by metal (silver and gold) sputtering, creating a microarray that enables the enrichment and identification of bacteria based on their unique Raman fingerprints. Testing the *P*-FS with various bacterial species (*E. coli* 25922, *S. aureus* 25923, *E. coli* MG1655, *Lactobacillus brevis*, and *S. mutans* 3065) demonstrated successful quantification through digitization analysis of SERS intensity mappings. The *P*-FS’s ability to enhance vibrational signatures, coupled with its scalable fabrication under routine conditions, positions it as a promising tool for reliable and ultrasensitive detection of various analytes, with strong potential to transition from laboratory research to clinical applications.

## Results and discussions

### Plasmonic membrane design and array fabrication

**Fig. 1:**
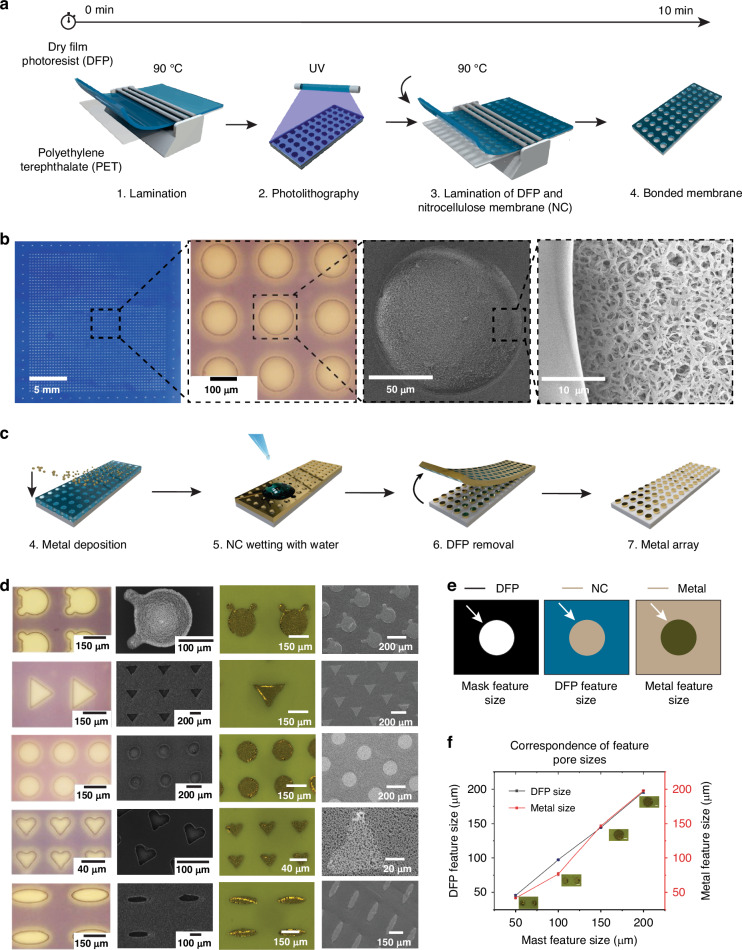
Fabrication and characterization of plasmonic membrane. **a** The process involves lamination of dry film photoresist (DFP) on polyethylene terephthalate (PET) at 90 °C, followed by photolithography using UV light, and then lamination of the patterned DFP onto a nitrocellulose (NC) membrane at 90 °C to produce the bonded membrane. **b** Visual and scanning electron microscope (SEM) images of the fabricated plasmonic membrane. The leftmost image shows the entire membrane with a scale bar of 5 mm. The subsequent images show magnified views of the patterned features with scale bars of 100 µm, 50 µm, and 10 µm, highlighting the precise pattern transfer and surface morphology. **c** Steps for creating the plasmonic array on the plasmonic membrane. Deposition of 10 nm thickness of metal on the pattern, wetting the NC with water, and removal of DFP to create the final plasmonic array. **d** Optical and SEM images of various shaped patterns (circles, triangles, squares, and custom shapes) on the DFP, NC, and metal layers, with corresponding scale bars ranging from 40 µm to 200 µm. **e** Diagram showing the correspondence between the mask feature size, DFP feature size, and metal feature size, indicating the fidelity of pattern transfer through the fabrication process. **f** Graph illustrating the correlation between mask feature size and the resulting DFP and metalized feature sizes. Insets show the corresponding images of the features at different stages of fabrication, with a scale for both DFP and metal sizes. Scale bar 50 µm. Data represent mean ± s.d.; *n* = 3 independent experiments

We demonstrated bacterial identification using a *P*-FS, where the nitrocellulose (NC) membrane was modified with a plasmonic array made of gold, and silver. The microarray was generated through desired shapes and sizes of designed patterns, which were transferred onto dry film photoresist (DFP) using UV light radiation during photolithography. The process begins with the lamination of DFP onto a polyethylene terephthalate (PET) substrate at 90 °C (Figs. 1a and S1), followed by photolithography where the DFP is exposed to UV light through a photomask to create a patterned array. This is then laminated with a NC membrane at 90 °C, resulting in a bonded membrane. Figure 1b shows a detailed view of the 100 µm-sized pattern generated on the membrane through the photolithography process, with a closer look through the SEM revealing intricate fibrous and porous structures of the membrane. As the NC was layered with DFP, metal was deposited on the top side of the DFP using a sputtering process to generate the plasmonic array for SERS enhancement. After wetting the NC membrane with water to facilitate subsequent steps, the DFP was removed, leaving the patterned plasmonic array on the NC membrane and resulting in a final product that mirrors the original photomask pattern (Fig. 1c, steps 4–7).

A metal layer of two different thicknesses, 10 and 30 nm, was applied to pattern sizes of 50, 100, 150, and 200 µm to optimize hotspot generation in each metal deposition. After coating with metal and removing the DFP, plasmonic arrays consisting of 40,400, 10,200, 4489, and 2600 metalized features were generated on the surface of the NC membrane (Fig. S2A). Various patterned shapes, including circles, triangles, and hearts, are showcased in Fig. 1d, demonstrating the process’s versatility and precision. The first column displays the photomask patterns on the DFP features, while subsequent columns show bright field images of patterns on the NC, and the final metal features with SEM images after the removal of DFP at different magnifications, emphasizing the method’s capability to create diverse and accurate patterns. Figure 1e illustrates the correspondence between mask feature size, DFP feature size, and metal feature size using different colors to represent each stage (DFP in black, NC in blue, and Metal in brown). The graph in Figs. 1f and S2B plots DFP feature size against mask feature size and metal feature size, showing a linear relationship. This correlation indicates the process’s predictability and scalability, with inset images providing visual reference for various feature sizes.

**Fig. 2:**
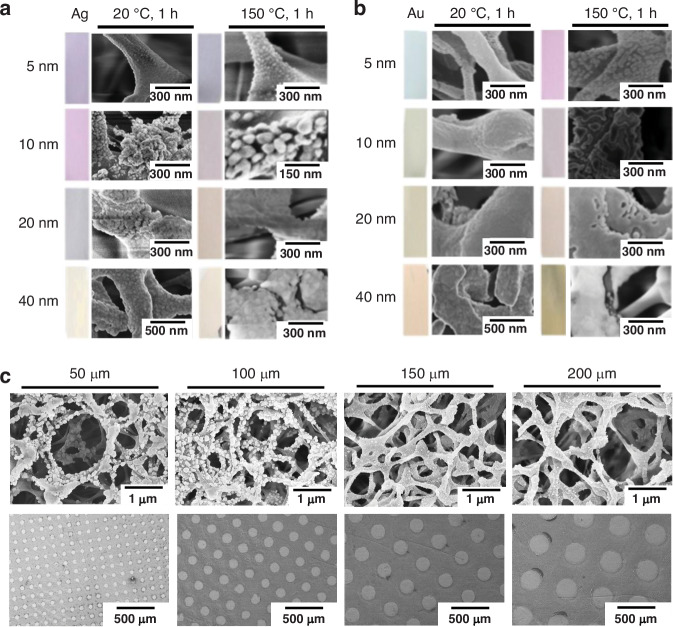
SEM characterization of metal-coated membrane. **a**, **b** SEM images showing the surface morphology of silver (Ag) and gold (Au) coated membranes at different thicknesses (5 nm, 10 nm, 20 nm, and 40 nm) and two different conditions: at 20 °C for 1 h (left column) and at 150 °C for 1 h (right column) with 100 µm metalized features. The scale bars represent 150 nm, 300 nm and 500 nm as indicated in each image. **c** SEM images illustrating the metalized features and patterned plasmonic arrays of the plasmonic membranes with different metalized features (50 µm, 100 µm, 150 µm, and 200 µm) at 5 nm thickness and 150 °C for 1 h. The top row shows high-magnification images with a scale bar of 1 µm, while the bottom row shows the patterned arrays with a scale bar of 500 µm

The objective of performing metal coating at different temperatures was to investigate the impact of temperature on the morphology of silver (Ag) and gold (Au) films, which directly affects on the SERS performance. Figure 2 presents SEM images illustrating the structural characteristics of Ag and Au films deposited at various thicknesses (5 nm, 10 nm, 20 nm, and 40 nm) and subjected to different thermal conditions (20 °C and 150 °C for 1 h each).

At 20 °C, the films generally appear smoother and more continuous, with granularity increasing with thickness. When heated to 150 °C, both Ag and Au films show significant morphological changes, including agglomeration and grain growth. This effect is more pronounced in thicker films and is particularly evident in Ag depositions. For instance, at 5 nm thickness, Ag shows noticeable agglomeration at 150 °C, while Au exhibits only slight changes. As the thickness increases, the degree of agglomeration and particle formation becomes more pronounced, especially for Ag films.

The final condition of 150 °C for 10 nm silver coating was chosen because it provided an optimal balance between surface roughness and the formation of effective SERS hotspots. At this condition, the Ag film forms distinct nanoparticles without excessive agglomeration, which is ideal for enhancing SERS signals. While Au films generally maintained a smoother morphology under the same conditions, Ag coatings showed superior SERS enhancement at 150 °C and 10 nm thickness.

These temperature-dependent structural changes in the metal coatings directly influence the surface characteristics of the membrane, which in turn affects its SERS performance. The study of these variations at different temperatures and thicknesses was crucial in determining the optimal conditions for our SERS applications.

Figure 2c shows the overall pattern and metalized features of the 50, 100, 150, and 200 µm plasmonic arrays on Ag deposition. The metalized features on the 50 µm plasmonic array reveal a dense network structure in the top micrograph, while for the 100 µm array show a similar dense network with clearly defined patterned metalized features and high roughness. In contrast, the 150 µm and 200 µm arrays display a consistent smooth network structure and the largest metalized feature, which, due to their high smoothness, did not generate hotspots for the SERS applications.

Overall, this demonstrates the process’s capability to create patterned membranes with various metalized features, maintaining uniformity and consistency even at different sizes, and highlights the precision and control of the fabrication process.

### Performance analysis of a hand-powered plasmonic fidget spinner

**Fig. 3:**
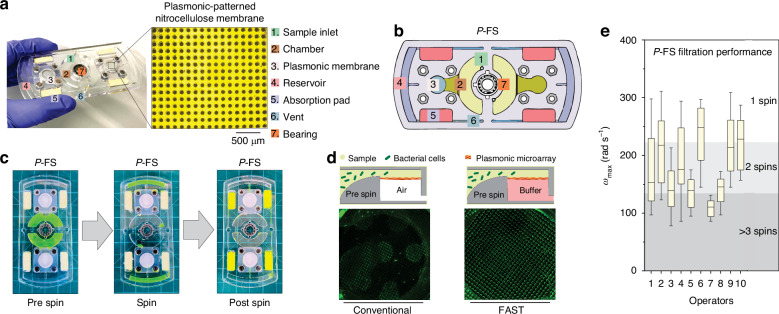
Model and operation of *P*-FS for bacterial enrichment. **a** Image of the *P*-FS device, highlighting the key components: (1) Sample inlet, (2) Chamber, (3) Plasmonic membrane, (4) Reservoir, (5) Absorption pad, (6) Vent, and (7) Bearing. An inset shows a magnified view of the plasmonic membrane. Scale bar 500 µm. **b** Schematic diagram of the *P*-FS device, color-coded to match the key components listed in (**a**). **c** Sequence showing the operation of the *P*-FS for spinning and enriching bacteria. The left image shows the device pre-spin, the middle image shows the device during spinning, and the right image shows the device post-spin. **d** Diagram illustrating the difference between two scenarios: (left) the conventional way where the chamber was filled with air; (right) before the spinning, the drainage chamber was filled with buffer creating uniform filtration across the membrane area that minimizes the hydrodynamic resistance during spinning and the distribution of bacteria was uniform on the membrane filter (FAST). **e** Scatter plot depicting the maximum angular velocity (*ω*_max_) achieved by ten different operators during spinning, measured in rad/s. The plot shows the distribution of *ω*_max_ across multiple trials, with one, two, and three or more spins indicated by different shades. Horizontal lines represent the average *ω*_max_ for each operator. (All operators, *n* = 10)

We developed a customized hand-powered *P*-FS that operates without electricity, and by replacing the NC membrane used in our Dx-FS study with an advanced plasmonic membrane, we successfully converted the Dx-FS into the *P*-FS. Figure 3 presents a comprehensive analysis of a microfluidic device designed for the enrichment and detection of bacterial cells using a plasmonic membrane, providing a schematic layout that indicates the position of each component to facilitate understanding of the fluid flow and interaction between the sample and the plasmonic membrane within the device.

Additionally, a photograph of the device is shown with labeled components, including the sample inlet (1), chamber (2), plasmonic membrane (3), reservoir (4), absorption pad (5), vent (6), and bearing (7) (Figs. 3a, b and S4). The close-up image highlights the detailed structure of the 14 mm plasmonic membrane, which is crucial for capturing and enriching bacteria from the sample (offset image Fig. 3a: white patterned on the blue background, Fig. S3). This membrane was fitted onto the *P*-FS for bacterial enrichment, with the detailed fabrication process provided in the methodology.

Figure 3c illustrates the operational workflow, showing the process of spinning to enrich bacteria, beginning with the device in its initial state, progressing through the spinning phase where the sample is moved towards the plasmonic membrane, and concluding with the post-spin state where the bacteria are concentrated on the membrane. In the pre-spin stage, a buffer was added to the bottom chamber of the plasmonic membrane using fluid-assisted separation technology (FAST), which ensured uniform distribution of bacterial cells on the membrane at the post-spin stage (Fig. 3d). FAST created a uniform pressure difference across the membrane by removing air from the chamber. During spinning, centrifugal force drove the sample towards the membrane, facilitating bacterial attachment, and post-spin, the bacteria were concentrated on the membrane, ready for detection.

The kinetic energy generated by hand was applied to the *P*-FS, converting it into centrifugal force that enabled the device to spin. Performance analysis revealed variability in spinning speeds achieved by different operators, similar to the operator-dependent variability observed in a Dx-FS device (Figs. 3e and S5). This variability suggests that the metal patterning on the filter did not affect the performance of filtration speed, possibly due to the nanoscale size of the patterning, which maintained consistent filtration efficiency across different spinning speeds. During spinning, an angular velocity (ω) of up to 311 rad s⁻¹ was generated, depending on the strength of the individual operator, and the filtration efficiency was directly related to the spinning power. We allowed 10 different operators to spin the device and summarized the power output across multiple trials. The ω_max_ range varied from 78 to 311 rad s⁻¹ across 100 measurements, with an average of 178.2 ± 62.2 rad s⁻¹.

**Fig. 4:**
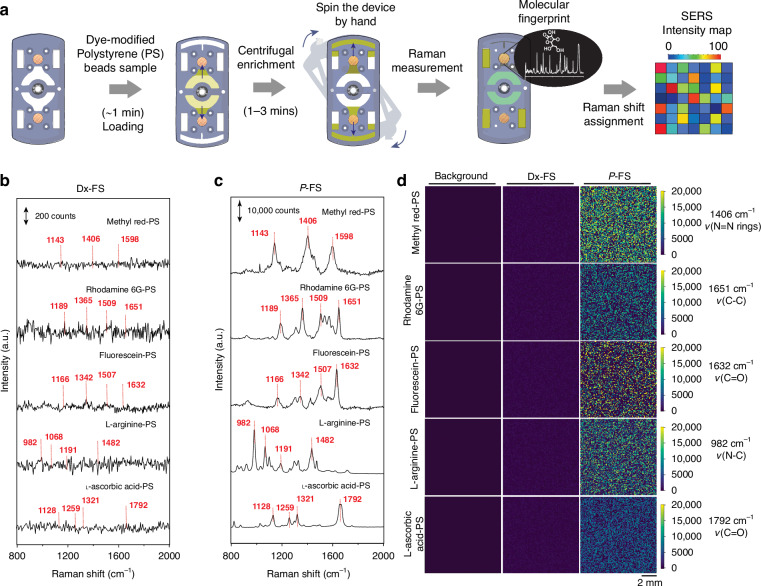
Operation and Raman signal analysis of molecules using *P*-FS. **a** Schematic of the operational workflow for the *P*-FS. The steps include loading a PS beads sample (~1 min), spinning the device by hand to achieve centrifugal enrichment (1–3 mins) and arrows (blue) indicate the movement of fluid, conducting Raman measurements to obtain molecular fingerprints, and assigning Raman shifts to create a SERS intensity map. **b** Raman spectra of various molecules-modified PS beads (Methyl red-PS, Rhodamine 6G-PS, Fluorescein-PS, L-arginine-PS, L-ascorbic acid-PS) obtained using the Dx-FS^23^. The intensity counts are shown up to 200 counts, with key Raman shift peaks labeled (e.g., 1143 cm⁻¹, 1406 cm⁻¹, 1598 cm⁻¹ for Methyl red-PS. **c** Enhanced Raman spectra of the same dye-modified PS beads obtained using the *P*-FS. The intensity counts are shown up to 10,000 counts, with the same key Raman shift peaks labeled as in (**b**), demonstrating significantly higher signal intensities. **d** SERS intensity maps comparing the background, original Dx-FS, and *P*-FS for each type of dye-modified PS bead. Specific Raman shifts corresponding to molecular vibrations (e.g., 1406 cm⁻¹ for ν(N=N rings), 1651 cm⁻¹ for ν(C=C), 1632 cm⁻¹ for ν(C=O)) are highlighted to show the enhancement in signal and spatial distribution of the Raman-active sites on the *P*-FS. Scale bar 2 mm

Raman spectroscopy was employed to identify distinct vibrational signatures in molecules by analyzing three dye-modified polystyrene (PS) beads coated with methyl red, rhodamine 6G, and fluorescein, as well as two biomolecule-modified PS beads with L-arginine and L-ascorbic acid. The workflow for the Raman measurement process, as outlined in Fig. 4a, includes loading the dye-modified PS bead sample into the device, spinning the device by hand for centrifugal enrichment, conducting Raman measurements to obtain molecular fingerprints, and generating a SERS intensity map. The beads enriched on the membrane were characterized using SERS spectra under specific measurement conditions: a 525 nm laser, 1-s exposure time, and 0.9 mW laser power. This setup demonstrated the stretching and bending vibrations of different bonds within the individual molecules. Comparing the Raman spectra obtained with the Dx-FS and *P*-FS from the different molecules highlighted a significant improvement in sensitivity (Fig. 4b, c). The *P*-FS provided clearer and stronger signals with specific molecular fingerprints for each dye, with key enhanced peaks observed at 1406 cm⁻¹ for methyl red-PS (N=N rings), 1651 cm⁻¹ for rhodamine 6G-PS (C–C), 1632 cm⁻¹ for fluorescein-PS (C–O), 982 cm⁻¹ for L-arginine-PS (N–C), and 1792 cm⁻¹ for L-ascorbic acid-PS (C=O), corresponding to their molecular vibrations (Fig. S6). The SERS intensity map demonstrated a significant enhancement—3.8-fold for the gold membrane and 32.7-fold for the silver membrane in the *P*-FS—compared to the control study using an NC membrane (Fig. S7).

Additionally, the study explored the effect of metal type (silver and gold), film thickness (5 nm, 10 nm, 20 nm, and 40 nm), and temperature (20 °C and 150 °C) on SERS intensity at specific vibrational modes for various dye-modified PS beads, including methyl red (1406 cm⁻¹, ν(N=N rings)), rhodamine 6G (1651 cm⁻¹, ν(C–C rings)), fluorescein (1632 cm⁻¹, ν(C–O rings)), L-arginine (982 cm⁻¹, ν(N–C rings)), and L-ascorbic acid (1792 cm⁻¹, ν(C=O rings)). These results highlight the importance of optimizing film thickness and temperature for effective SERS-based detection, with significant differences observed between Ag and Au films (Fig. S8). The optimal SERS signals were achieved with silver at a thickness of 10 nm and a temperature of 150 °C. Further analysis of different silver pattern sizes (50 µm, 100 µm, 150 µm, and 200 µm) revealed that the best plasmonic signal was obtained with a 100 µm pattern size, which was selected for further applications (Fig. S9). For all the molecules, at their main peak positions—1406 cm⁻¹ for methyl red, 1651 cm⁻¹ for rhodamine 6G, 1632 cm⁻¹ for fluorescein, 982 cm⁻¹ for L-arginine, and 1792 cm⁻¹ for L-ascorbic acid—Raman intensity measurements across 10,000 metalized feature (153.94 mm²) confirmed the presence of the molecules (Fig. 4d).

**Fig. 5:**
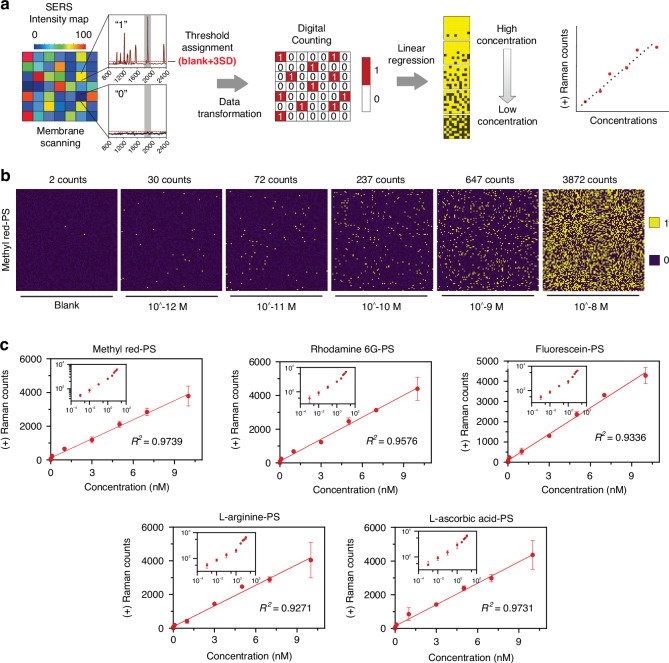
Digital mapping and quantification of dye modified PS beads using SERS. **a** Schematic of the digital counting process using SERS intensity maps. Membrane scanning generates intensity maps where thresholds are assigned to distinguish positive (“1”) from negative (“0”) Raman signals. Data transformation converts the intensity map into a binary matrix for digital counting. Linear regression is then applied to correlate Raman counts with concentrations, allowing for the differentiation between high and low concentrations of analytes. **b** SERS intensity maps of methyl red-PS beads at various concentrations, ranging from blank to 10⁻⁸ M. The number of positive counts (yellow) increases with higher concentrations, illustrating the sensitivity of the detection method. **c** Calibration curves for different molecule-modified PS beads: Methyl red-PS, Rhodamine 6G-PS, Fluorescein-PS, L-arginine-PS, and L-ascorbic acid-PS. Each plot shows the correlation between Raman counts and bead concentrations. An inset graph plotted using a logarithmic scale to cover the entire concentration range. Data represent mean ± s.d.; *n* = 3 digital counting batches

Based on the SERS intensity, all metalized feature (~10,000, 153.94 mm²) present in the membrane were mapped to determine a threshold (blank + 3 × standard deviation) for distinguishing the presence of molecules, which was then used to differentiate between positive (“1”) and negative (“0”) Raman signals, transforming the data into a binary format. This binary data was subsequently used for digital counting, followed by linear regression analysis to correlate Raman counts with dye concentrations, where higher concentrations yielded higher Raman counts, establishing a linear relationship between signals and analyte concentration (Fig. 5a).

Here, we present SERS intensity digital counting for methyl red-PS corresponding to a specific Raman shift (1406 cm⁻¹), with signal counts increasing as concentrations ranged from 0 to 10⁻⁸ M. These Raman counts visually demonstrate the correlation between concentration and detected Raman signals, where higher concentrations result in denser and more numerous signal “1” areas (represented in yellow), while “0” areas, which did not cross the threshold (532.5), are shown in purple (Fig. 5b). Figure 5c presents linear regression analyses for various dye-modified PS beads, including methyl red-PS, rhodamine 6G-PS, fluorescein-PS, L-arginine-PS, and L-ascorbic acid-PS, with each graph plotting the number of positive Raman counts against the concentration of dye-PS beads, and insets highlighting the correlations at lower concentrations. The high *R*² values (0.9897 for methyl red-PS, 0.9919 for rhodamine 6G-PS, 0.9934 for fluorescein-PS, 0.9848 for L-arginine-PS, and 0.9736 for L-ascorbic acid-PS) indicate a strong linear relationship, confirming the reliability and sensitivity of the digital counting method in quantifying low concentrations of dye-modified PS beads. The calculated limits for blank (LOB = Mean of Blank + 1.645 × S.D. of Blank), detection (LOD = LOB + 3.3 × S.D. of Blank), and quantification (LOQ = Mean of Blank + 10 × S.D. of Blank) for methyl red-PS were 6.2 pM, 12 pM, and 38 pM, (*y* = 4 × 10^11^*x* + 2.3) respectively; for rhodamine 6G-PS were 4.1 pM, 8.2 pM, and 25 pM, (*y* = 4 × 10^11^*x* + 3) respectively; for fluorescein-PS were 4.7 pM, 9.5 pM, and 28 pM, (*y* = 4 × 10^11^*x* + 1.7) respectively; for L-arginine-PS were 4.1 pM, 8.2 pM, and 25 pM, (*y* = 4 × 10^11^*x* + 2) respectively; and for L-ascorbic acid-PS were 5.0 pM, 10.0 pM, and 30.5 pM, (*y* = 4 × 10^11^*x* + 1.7) respectively. Overall, the process flow, intensity maps, and regression analyses in this figure collectively validate the SERS-based approach as a robust and sensitive technique for detecting and quantifying low concentrations of analytes. The use of an array of metalized features on the membrane plays a crucial role in converting Raman intensity to digital counts, which further enhances the sensitivity of the system. By concentrating the electromagnetic field into localized hotspots, the patterned array produces more distinct and intense Raman signals, which can be accurately digitized. Additionally, metalizing the entire membrane could interfere with the critical function of retaining the filtration and flow properties necessary for the effective collection and trapping of bacterial cells. The method’s strength lies in its ability to visually represent and quantify the relationship between analyte concentration and Raman signal intensity, providing both qualitative and quantitative insights.

### Identification of bacteria using Raman spectroscopy

We explored the potential of the *P*-FS to identify bacterial species by analyzing the unique molecular compositions that define different bacterial phenotypes, which result in distinct variations in their Raman spectra. Each bacterial species exhibits a characteristic set of vibrational modes in its Raman spectrum, reflecting the specific biomolecular makeup of its cell structure, such as proteins, lipids, nucleic acids, and carbohydrates. By harnessing these unique spectral signatures, the *P*-FS allows for the precise identification of bacterial species based on their distinct Raman fingerprints, enabling differentiation even among closely related bacterial strains. In this study, we employed five different bacterial strains—*E. coli* 25922, *S. aureus* 25923, *E. coli* MG1655, *Lactobacillus brevis*, and *S. mutans* 3065—each at a concentration of 10⁵ CFU/mL, for identification using the SERS mechanism (Table S1).

**Fig. 6:**
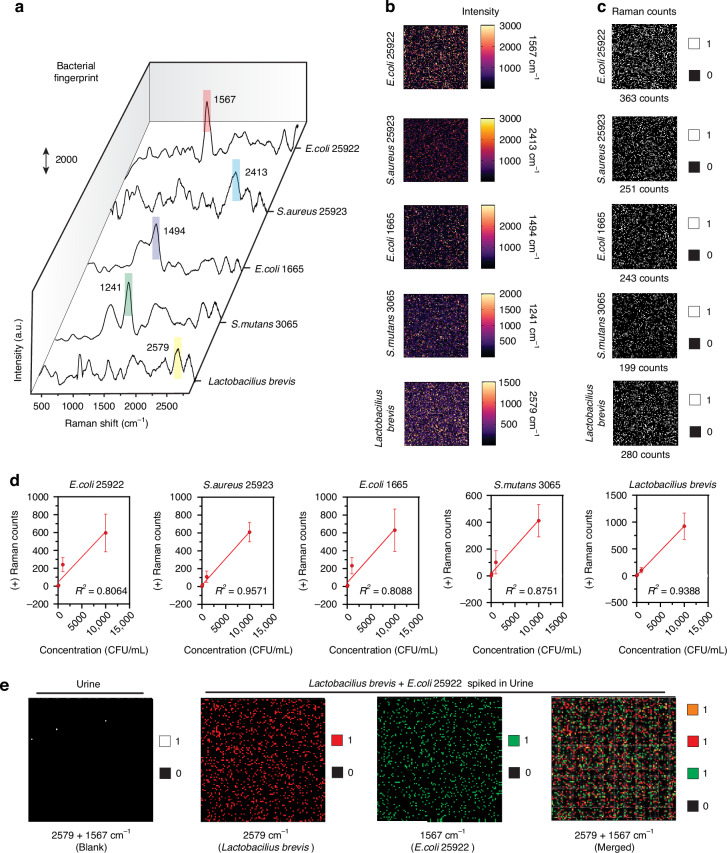
Identification and digital counting of bacterial species using SERS. **a** Raman spectra of various bacterial species highlighting key peaks: *E. coli* 25922 (1567 cm⁻¹), *S. aureus* 25923 (2413 cm⁻¹), *E. coli* 1655 (1494 cm⁻¹), *S. mutans* 3065 (1241 cm⁻¹), and *Lactobacillus brevis* (2579 cm⁻¹). **b** SERS intensity maps for the respective bacterial species at the indicated Raman shifts. The color scale indicates Raman intensity, with warmer colors representing higher intensities. **c** Digital counting maps showing the binary presence (1) or absence (0) of Raman signals for each bacterial species. The total count of positive detections is listed for each species. **d** Calibration curves showing the correlation between Raman counts and bacterial concentration for each species: *E. coli* 25922, *S. aureus* 25923, *E. coli* 1665, *S. mutans* 3065, and *Lactobacillus brevis*. The *R*² values indicate the linearity of the response. Data represent mean ± s.d.; *n* = 3 independent experiments. **e** SERS intensity maps of urine samples spiked with Lactobacillus brevis and *E. coli* 25922. The leftmost map shows a blank urine sample. The middle maps show the detection of *Lactobacillus brevis* (2579 cm⁻¹) and *E. coli* 25922 (1567 cm⁻¹) individually, while the rightmost map shows the merged detection of both bacteria in the same sample

A 1 mL bacterial solution was introduced into individual metalized features of the *P*-FS device, where the bacteria were enriched on the plasmonic membrane array during the spinning process. Following enrichment, the bacteria on the plasmonic membrane were characterized using Raman spectroscopy, which provided detailed spectral fingerprints unique to each bacterial species (Fig. 6a). The distribution of bacterial cells on the plasmonic array was modeled using a Poisson distribution at this concentration, which predicted that more than 90% of the plasmonic arrays contained between 1 and 9 bacterial cells (Fig. S10). This uniform distribution was achieved because FAST created a uniform pressure difference across the membrane during spinning, allowing centrifugal force to effectively drive the sample toward the membrane, facilitating consistent bacterial distribution across the plasmonic arrays^24^.

For each bacterial species, we mapped 10,000 metalized features, each measuring 100 × 100 µm², on the plasmonic membrane. The bacteria exhibited characteristic Raman spectra with high signal-to-noise ratios, achieved with an integration time of 30 s and a 532 nm laser at a power of 0.99 mW. These spectra provided some shared basic structural components such as DNA (adenine 726 cm^−1^), cell wall (957 cm^−1^) and proteins (1003 cm^−1^ for C-H plane of phenylalanine and C-C aromatic ring breathing) and many distinct vibrational signatures for each bacterial species, enabling their precise identification and differentiation^25^. Key vibrational modes unique to each bacterial species were identified and assigned, including 1567 cm⁻¹ for *E. coli* 25922, 2413 cm⁻¹ for *S. aureus* 25923, 1494 cm⁻¹ for *E. coli* MG1655, 1241 cm⁻¹ for *S. mutans* 3065, and 2579 cm⁻¹ for *Lactobacillus brevis*. These distinct peaks, corresponding to specific molecular vibrations unique to each bacterial species, allow for accurate quantification and are visually represented in SERS intensity maps that depict the distribution and density of Raman signals, with higher intensity regions highlighting bacterial fingerprints aligned with the specific Raman shifts identified in the spectra (Fig. 6b).

After assigning the threshold on intensity of each Raman shift for individual bacteria (498.47 at 1567 cm⁻¹ for *E. coli* 25922, 513.23 at 2413 cm⁻¹ for *S. aureus* 25923, 508 at 1494 cm⁻¹ for *E. coli* MG1655, 521.27 at 1241 cm⁻¹ for *S. mutans* 3065, and 511 at 2579 cm⁻¹ for *Lactobacillus brevis*) these intensity maps are transformed into binary data, assigning “1” for the presence and “0” for the absence of signals. The total Raman counts for each bacterial species were recorded, demonstrating the effectiveness of digital counting in quantifying bacterial presence: *E. coli* 25922 had 363 counts, *S. aureus* 25923 had 251 counts, *E. coli* MG1655 had 243 counts, *S. mutans* 3065 had 199 counts, and *Lactobacillus brevis* had 280 counts (Fig. 6c).

The SERS intensity maps and digital counting results effectively illustrate the ability to detect and quantify bacterial presence. Transforming intensity maps into binary data facilitates straightforward quantification, providing clear and interpretable results. Linear regression analyses in Fig. 6d show strong correlations between Raman counts and bacterial concentrations for all tested species, with high *R*² values confirming the method’s accuracy. The calculated LOB, LOD, and LOQ were 15, 31, and 94 CFU/mL for *E. coli* 25922; 26, 53, and 163 CFU/mL for *S. aureus* 25923; 14, 29, and 89 CFU/mL for *E. coli* MG1655; 22, 45, and 138 CFU/mL for *S. mutans* 3065; and 20, 41, and 124 CFU/mL for *Lactobacillus brevis*, respectively, where S.D. represents the standard deviation. This quantitative capability is essential for applications requiring precise bacterial load measurements.

Next, we conducted a comparative study with multiple metalized features (50 µm, 100 µm, 150 µm, and 200 µm) to establish a clearer understanding of their impact on detection sensitivity. As shown in the Fig. S11, the data indicate that the 100 µm metalized feature offers the best LOD, likely due to enhanced plasmonic hotspot formation that significantly amplifies the Raman signal, leading to better detection capabilities. These results demonstrate that the choice of a 100 µm metalized feature provides a favorable compromise between trapping efficiency and SERS enhancement, leading to the lowest LOD and the most sensitive detection.

Next, we wanted to demonstrate the detection capability of the *P*-FS in complex biological samples, such as urine, where multiple bacterial species may be present. Figure 6e in the image presents digital counting results of urine samples that have been spiked with *Lactobacillus brevis* and *E. coli* 25922. The first map on the left serves as a control, showing the SERS intensity map of a blank urine sample with negligible signal, indicating the absence of detectable bacterial fingerprints. The second map illustrates the detection of *Lactobacillus brevis* in urine, with red dots representing the presence of the bacteria at a Raman shift of 2579 cm⁻¹. The third map shows the detection of *E. coli* 25922, where green dots indicate bacterial presence at a Raman shift of 1567 cm⁻¹. The rightmost map merges the detection of both *Lactobacillus brevis* and *E. coli* 25922 in the same urine sample, with red and green dots clearly identifying each species. This visualization underscores the SERS method’s ability to simultaneously detect and distinguish multiple bacterial species within a single sample, demonstrating its specificity and potential for clinical applications, such as diagnosing infections in urine samples. Next, the ability of the *P*-FS integrated with SERS to detect and quantify closely related *E. coli* strains—MG1655 and 25922—in mixed populations (Fig. S12A). The SERS intensity maps showed the individual and combined detection of the two strains, highlighting their unique spectral signatures (1494 cm⁻¹ for MG1655 and 1567 cm⁻¹ for 25922). However, the close proximity of these peaks presents a challenge in distinguishing the strains, particularly at lower concentrations. The calibration curves reveal a linear relationship between the actual and estimated concentrations, demonstrating the feasibility of quantification through digital counting, though with notable variability due to signal overlap (Fig. S12B). The accuracy heatmaps further indicate that detection accuracy is high at higher concentrations but decreases significantly at lower concentrations due to the challenges of weak signal intensities and peak overlap (Fig. S12C). These findings underscore the potential of the *P*-FS for bacterial detection in mixed populations, while also highlighting the need for further optimization to improve accuracy in complex and low-concentration samples.

### Outlook

This study presents an innovative method for the detection, identification, and quantification of bacterial species by combining Raman spectroscopy, digital counting, and plasmonic-enhanced devices. The microfluidic device, featuring a plasmonic membrane, effectively enriches bacterial cells through a simple hand-spinning process, enhancing both user-friendliness and broad applicability. The *P*-FS significantly improves the sensitivity of Raman signal detection, enabling the detection of low concentrations of bacterial cells and other analytes—an essential feature for high-sensitivity applications.

The use of an array of metalized features on the membrane, rather than metalizing the entire surface, serves multiple critical functions. This design enables the conversion of Raman intensity to digital counts, enhancing detection sensitivity and precision. The patterned array creates localized electromagnetic hotspots that produce more distinct and intense Raman signals, allowing for effective detection even at low bacterial concentrations. Furthermore, this approach preserves the membrane’s essential filtration and flow properties, which are crucial for efficient bacterial collection and trapping. The strategic placement of hotspots in the array optimizes Raman signal enhancement across the membrane surface, balancing enhanced detection capabilities with maintained functionality of the NC substrate. This design choice is fundamental to the *P*-FS system’s ability to combine sensitive detection with practical sample handling.

Unique Raman fingerprints were identified for different bacterial species, allowing precise identification based on distinct Raman shifts and facilitating accurate microbial analysis. The digital counting method transforms SERS intensity maps into binary data, enabling straightforward quantification. Linear regression analyses demonstrated strong correlations between Raman counts and bacterial concentrations, confirming the method’s accuracy and reliability for quantitative measurements. The study also applied this method to detect bacterial species in complex samples, such as urine, highlighting its practical utility in real-world diagnostic scenarios.

Whether a spot represented a single bacterium or a cluster of multiple bacteria, it was counted equivalently as “1.” While this approach simplifies the analysis, it also introduces a potential limitation in distinguishing between individual and clustered bacteria. Our approach demonstrates high sensitivity and specificity for bacterial detection, but we acknowledge that the current total time-to-result requires optimization for rapid diagnostics.

While the SERS detection itself can potentially occur within seconds to minutes as mentioned in the introduction, the entire process from sample loading to data acquisition currently takes approximately 3.3 h. This includes sample preparation steps such as loading (1 min), spinning (3 min), membrane removal (2 min), and air drying (10 min), followed by Raman scanning (3 h). Future improvements will focus on reducing this time, particularly by exploring portable Raman devices for faster scanning and optimizing the sample preparation process. These enhancements aim to make the technique more suitable for rapid infection identification in practical settings. The advancements in device design, signal enhancement, and data processing pave the way for numerous future applications in scientific and industrial fields, offering a powerful tool for microbial analysis and beyond. Future developments, particularly integrating SERS in the *P*-FS as a point-of-care test, hold promise for enhancing its reliability and accessibility in remote and developing areas, positioning it as a revolutionary tool in microbial diagnostics and public health.

## Materials and methods

### Chemicals

In this study, we utilized a variety of materials and reagents to conduct our experiments. The dry film photoresist (40 μm thick, cat# FL-40600-150M) was obtained from FORTEX, while the polyethylene terephthalate (thickness 2 mm, cat# GF14511215) was sourced from Sigma. The nitrocellulose membrane (cat# 1620115) was supplied by Bio-Rad, and the polycarbonate sheet was acquired from I-components, Co., Ltd. We used a double-sided adhesive layer (DFM 200 clear150 POLY H-9 V-95) from FLEXcon. Ethanol was provided by Duksan Pure Chemicals, and various culture media, including Luria Bertani (LB) broth, Mueller Hinton (MH) broth, LB agar, brain heart infusion medium (BHI), Lactobacilli MRS agar, and Lactobacilli MRS broth, were all sourced from DifcoTM Generic Labware. Ampicillin (100 mg/mL) was obtained from Thermo Fisher. Additionally, we used a range of chemicals from Sigma-Aldrich, including sucrose (cat# S7903), methyl red (cat# 250198 and cat# 114510), rhodamine B (cat# 83689), fluorescein (cat# 2321-07-5), and rhodamine 6G (cat# R4127). Phosphate-buffered saline (PBS) was supplied by Thermo Fisher Scientific, and hydrochloric acid (cat# 7647-01-0) was sourced from Samchun. These materials and reagents were integral to the preparation and execution of the experimental procedures in this study.

### Instruments

UV-Vis spectra were measured using a spectrophotometer (TECAN, Morrisville, NC, USA). A laminator from Hyundae was used for the lamination processes, and photolithography was performed using the MIDAS MDA-400TS system. For coating procedures, a sputter coater (DC Sputter, SRN-120) was employed. The milling and cutting processes were conducted using the Promill Smart 3530 milling machine from Protek and the CE3000-60 cutting plotter from Graphtec, respectively. High-speed imaging was captured using the Phantom Miro 310 camera from Vision Research, while a servo motor (EDB2000-56V24/48-S) from ERAETECH provided precise mechanical movements. For detailed analysis, a confocal Raman microscope (alpha300R) from WITec and a field emission scanning electron microscope (SU7000) from Hitachi High-Tech were employed. a 532 nm laser (CPS532-C2) from Thorlabs, a light sensor (LX1972) and an Arduino controller (DFRduino UNO R3), both from DFROBOT, were utilized to monitor the rotations.

## Methodology

### Fabrication of plasmonic membrane

The fabrication of the plasmonic membrane involves a series of steps including photolithography and metal deposition. The process begins by laminating dry film photoresist (DFP) with a polyethylene terephthalate (PET) substrate. This lamination is performed at 90 °C, where the DFP and PET are sandwiched between two parallel plates to mold the DFP. The resulting film is then subjected to the photolithography process. During this step, the film is exposed to a mask pattern that imprints the desired design onto the DFP. Upon exposure to light, the photoresist crosslinks and polymerizes with the substrate, forming the microarray pattern. Following the photolithography process, the film is developed to reveal the microarray. The designed film is then laminated with a NC membrane at 90 °C to bond the layers. The unexposed areas of the photoresist were removed during the development process using a developer solution to achieve a micropattern. Once the micropattern is generated, a thin layer of silver, approximately 10 nm thick, is deposited onto the microarray using a sputtering process. This deposition is carried out in a 100 µm microarray configuration. To finalize the fabrication, the entire membrane is immersed in deionized (DI) water for 24 h to remove the DFP. After the DFP is removed, the membrane is dried for a few hours. The removal of the DFP leaves behind a structured array of metal on the NC membrane, thereby creating the plasmonic membrane. This plasmonic membrane is then ready for use in various applications, such as enhancing Raman signal detection in biosensing.

### Plasmonic fidget spinner design and fabrication

The plasmonic fidget spinner was designed based on previous work and comprises three main parts: the main body, middle layer, and top layer. The design process began by creating a three-dimensional digital model using computer-aided design (CAD) software. This digital design was then precisely carved onto a 5 mm thick polycarbonate sheet, which served as the main body of the device, using a computer-numerical-control (CNC) milling machine (Promill Smart 3530, Protek). A 0.1 mm thick double-sided adhesive layer was applied to attach the top layer, which was made from a 0.125 mm thick transparent polycarbonate sheet. This top layer was carefully cut to the required shape using a cutting plotter (CE3000-60, Graphtec). The entire unit was designed with dimensions of 106 mm in length, 50 mm in width, and 5.5 mm in thickness, containing three functional parts: a sample loading area, a filtration unit, and a reservoir. The filtration unit was assembled with a 14 mm plasmonic NC membrane, which is key to the device’s function. To ensure proper sealing and prevent leaks, the area underneath the filter chamber was closed with a rubber seal and a plastic cover equipped with an injection hole for sample introduction. The fabricated device was ergonomically designed to fit comfortably in the hands of both men and women, making it easy to use.

### Spin characterization and filtration rate

The spinning speed of the *P*-FS was characterized using a custom-built setup. This setup included a 532 nm laser (CPS532-C2, Thorlabs) and an Arduino controller (DFRduino UNO R3, DFROBOT) integrated with a light sensor. The *P*-FS was placed in the path of the laser, which emitted a light intensity of 6 kHz. As the *P*-FS spun, it intermittently blocked the laser beam, allowing the light intensity variations to be analyzed and measured to determine the spinning speed. Additionally, a high-speed camera was employed to capture detailed spinning dynamics, providing precise measurements of the device’s rotational behavior. To measure the flow rate of the *P*-FS, a home-built spinning platform was utilized, allowing for the determination of angular velocities. The *P*-FS was spun using a servo motor set to specific rotational frequencies. The servo motor was synchronized with the camera to capture images of the device at the same positions at a rate of 5 frames per second. The flow volume through the *P*-FS was then estimated based on the angular velocity, providing insights into the filtration rate and efficiency of the device.

### Cell culture and reagents

In this study, we focused on the cultivation and preparation of five bacterial strains: *Escherichia coli* 25922, *Staphylococcus aureus* 25923, *Escherichia coli* MG1665, *Lactobacillus brevis*, and *Streptococcus mutans* 3065. Below are the detailed procedures for culturing each of these strains (Table S1):

Culturing *Escherichia coli* 25922 and *Staphylococcus aureus* 25923: *E. coli* 25922 and *S. aureus* 25923 were initially streaked from their respective stock cultures onto separate LB agar plates to isolate individual colonies. The plates were incubated at 37 °C for 18 h in a bacterial incubator. This incubation period allowed the bacteria to grow and form visible colonies on the agar surface. After incubation, a single isolated colony from each plate was selected and transferred into a tube containing LB broth. The cultures were incubated at 37 °C with constant shaking at 200 rpm for 12 h to ensure optimal bacterial growth and to reach the desired bacterial density. For *E. coli* MG1665, which contains an ampicillin resistance gene, the LB media was supplemented with 100 µg/mL of ampicillin. This selective pressure ensured the maintenance of the plasmid and the survival of only the resistant bacteria. The culture conditions were identical to those used for *E. coli* 25922 and *S. aureus* 25923.

Culturing *Lactobacillus brevis*: *L. brevis* was streaked from its stock culture onto Lactobacilli MRS (de Man, Rogosa, and Sharpe) agar, which is specifically formulated to support the growth of lactobacilli by providing the necessary nutrients and pH conditions. The streaked MRS agar plates were incubated at 30 °C for 48 h. The lower temperature and extended incubation period are tailored to the slower growth rate and optimal temperature range of *L. brevis*. After 48 h, an isolated colony was transferred into Lactobacilli MRS broth and incubated at 30 °C with gentle shaking for 48 h. This prolonged incubation ensures the culture reaches a sufficient density for downstream applications.

Culturing *Streptococcus mutans* 3065: *S. mutans* 3065 was streaked onto brain heart infusion (BHI) agar supplemented with 1% sucrose. This medium is rich in nutrients, providing an ideal environment for the growth of *S. mutans*, a facultative anaerobe that thrives in nutrient-rich conditions. The plates were incubated at 37 °C in a 5% CO₂ atmosphere, mimicking the anaerobic conditions similar to the oral cavity where *S. mutans* naturally resides. After 24–48 h, visible colonies were formed. An isolated colony was then inoculated into BHI broth supplemented with 1% sucrose and incubated under the same conditions. This setup allowed for the consistent growth of *S. mutans* for subsequent use.

Harvesting and preparation of bacterial cultures: after the incubation period, the bacterial cultures were transferred into centrifuge tubes and centrifuged at 3000 rpm for 15 min. This step was performed to pellet the bacterial cells at the bottom of the tubes, separating them from the liquid culture medium. The bacterial pellets were carefully resuspended in 1× PBS (phosphate-buffered saline) to maintain the cells in a stable, isotonic environment, suitable for further experimental procedures. Resuspension also facilitated accurate counting and further handling of the bacterial samples. The bacterial concentration in the resuspended solution was determined using a hemocytometer, a device that allows for counting cells under a microscope. Additionally, the optical density (OD) of the suspension was measured at 620 nm using a spectrophotometer to estimate the bacterial concentration. Based on these measurements, the bacterial concentration was adjusted to 1 × 10⁵ CFU/mL by appropriate dilution with PBS. This standardized concentration was essential for ensuring consistency across experiments.

### SERS analysis through plasmonic membrane

To perform SERS analysis, 1 mL of dye-modified polystyrene beads (PS) with a diameter of 1 µm and varying concentrations were introduced into the metalized features of the *P*-FS. The device was spun until the entire sample had been filtered through the membrane, typically requiring more than three spins. The beads that remained on the plasmonic membrane after filtration were used as residues for further analysis. The plasmonic membrane was carefully removed from the fidget spinner and allowed to dry before being subjected to Raman signaling. SERS analysis was conducted using a confocal Raman microscope (alpha300R, WITec) with a 525 nm diode laser operating at 0.9 mW. The excitation light was focused onto the plasmonic membrane using a 50×/0.8 NA objective lens with ten accumulations for enhanced signal detection. From each plasmonic membrane, 20 random metalized features were selected to determine the number of beads present. The spectral variations generated by each isolate allowed for their identification and differentiation. The raw spectra were normalized to their highest points using OriginPro software for accurate analysis.

### Bacterial analysis through SERS

For bacterial analysis, 10⁵ CFU/mL of each bacterial species (*Escherichia coli* 25922, *Staphylococcus aureus* 25923, *Escherichia coli* MG 1665, *Lactobacillus brevis*, and *Streptococcus mutans*), as well as a mixed population of *E. coli* 25922 and *L. brevis*, were spiked into 1 mL of urine. The urine samples were then transferred to the *P*-FS chamber and subjected to spinning to enrich the bacteria on the membrane. The enriched bacteria were characterized using SERS. The plasmonic membrane was allowed to dry before Raman signaling. Using the alpha300R confocal Raman microscope, Raman signals were obtained from the bacteria using a 532 nm diode laser at 0.99 mW, focused with a 50×/0.8 NA objective lens and ten accumulations. Twenty different metalized feature were selected based on the focus points to generate spectral intensity data for the bacteria. Specific biological and spectral variations were observed, allowing for the differentiation of bacterial species. In the mixed population, distinct spectral signatures assignment from the same plasmonic membrane indicated that the bacteria could be distinguished, enabling identification of mixed bacterial populations from the samples.

### SERS digitization

Raman signal intensities generated from different PS beads and bacterial samples were compared against the intensity of a blank sample to establish a threshold. The intensity from each metalized feature was then converted into binary digits based on this threshold: “0” for signals below the threshold (considered negative) and “1” for signals exceeding the threshold (considered positive). The threshold value was calculated using the following formula:

$${\rm{Threshold}}={\rm{Intensity\; of\; blank}}+3{\rm{S}}.{\rm{D}}.$$

This approach enabled the digitization of SERS data for straightforward interpretation and analysis.

### SEM imaging

The surface morphology of the plasmonic membrane after filtration was characterized using scanning electron microscopy (SEM). The plasmonic membrane was air-dried and then examined using a field emission scanning electron microscope (SU7000, Hitachi) at an acceleration voltage of 10 kV and a working distance of 6 mm. SEM imaging provided detailed visualization of the surface structure and the distribution of particles on the plasmonic membrane, offering insights into the filtration process and the interaction of beads and bacteria with the membrane surface.

### Statistics and reproducibility

Data were plotted and analyzed using Microsoft Excel, Origin Software, and GraphPad Prism 9.3.1. Bar and line plots are represented as mean ± standard deviation (SD). For data displayed in boxes (box-and-whisker plots), the dots show individual values, and the whiskers show minimal and maximal values of each data point. Two-group comparisons were performed using a two-tailed unpaired Student’s *t* test, and three- or more group comparisons were conducted using one-way ANOVA. All data represent mean ± SD.

## Supplementary information

Supporting Information

## Data Availability

Data used to generate results in the current study are available from the corresponding author upon reasonable request.

## References

[1] Lee, W. et al. A fully integrated bacterial pathogen detection system based on count-on-a-cartridge platform for rapid, ultrasensitive, highly accurate and culture-free assay. *Biosens. Bioelectron.* **152**, 112007 (2020). 31941616 10.1016/j.bios.2020.112007

[2] Dietvorst, J., Vilaplana, L., Uria, N., Marco, M. P. & Muñoz-Berbel, X. Current and near-future technologies for antibiotic susceptibility testing and resistant bacteria detection. *Trends Anal. Chem.* **127**, 115891 (2020).

[3] Kamalrathne, T., Amaratunga, D., Haigh, R. & Kodituwakku, L. Need for effective detection and early warnings for epidemic and pandemic preparedness planning in the context of multi-hazards: lessons from the COVID-19 pandemic. *Int. J. Disaster Risk Reduct.* **92**, 103724 (2023). 37197332 10.1016/j.ijdrr.2023.103724 PMC10148710

[4] Franco-Duarte, R. et al. Advances in chemical and biological methods to identify microorganisms—from past to present. *Microorganisms* **7**, 130 (2019). 31086084 10.3390/microorganisms7050130 PMC6560418

[5] Sil, S., Mukherjee, R., Kumar, N. & Umapathy, S. Potential and challenges of pathogen detection using Raman spectroscopy. *Biomed. Spectrosc. Microsc. Imaging SPIE* **19**, 31 (2020).

[6] Strola, S. A. et al. Single bacteria identification by Raman spectroscopy. *J. Biomed. Opt.* **19**, 111610 (2014). 25028774 10.1117/1.JBO.19.11.111610

[7] Kumar, S. et al. Myoglobin and polydopamine-engineered Raman nanoprobes for detecting, imaging, and monitoring reactive oxygen species in biological samples and living cells. *Small* **13**, 1701584 (2017). 10.1002/smll.201701584 28902980

[8] Qian, Y. et al. Combined SERS microfluidic chip with gold nanocone array for effective early lung cancer prognosis in mice model. *Int. J. Nanomed.* **18**, 3429–3442 (2023). 10.2147/IJN.S411395 PMC10295598 37383221

[9] Cao, X. et al. A pump-free and high-throughput microfluidic chip for highly sensitive SERS assay of gastric cancer-related circulating tumor DNA via a cascade signal amplification strategy. *J. Nanobiotechnol.* **20**, 271 (2022). 10.1186/s12951-022-01481-y PMC9188168 35690820

[10] Wang, L. et al. Applications of Raman spectroscopy in bacterial infections: principles, advantages, and shortcomings. *Front. Microbiol.* **12**, 683580 (2021). 34349740 10.3389/fmicb.2021.683580 PMC8327204

[11] Dina, N. et al. Rapid single-cell detection and identification of pathogens by using surface-enhanced Raman spectroscopy. *Analyst* **142**, 1782–1789 (2017). 28430277 10.1039/c7an00106a

[12] Pistiki, A. et al. Comparison of different label-free Raman spectroscopy approaches for the discrimination of clinical MRSA and MSSA isolates. *Microbiol. Spectr.* **10**, e00763–e00822 (2022). 10.1128/spectrum.00763-22 PMC9603629 36005817

[13] Bi, X. et al. Digital colloid-enhanced Raman spectroscopy by single-molecule counting. *Nature* **628**, 771–775 (2024). 38632399 10.1038/s41586-024-07218-1

[14] Liu, Y. et al. Digital plasmonic nanobubble detection for rapid and ultrasensitive virus diagnostics. *Nat. Commun.* **13**, 1687 (2022). 35354801 10.1038/s41467-022-29025-w PMC8967834

[15] Zhang, Y. et al. General approach to surface-accessible plasmonic Pickering emulsions for SERS sensing and interfacial catalysis. *Nat. Commun.* **14**, 1392 (2023). 36914627 10.1038/s41467-023-37001-1 PMC10011407

[16] Schorr, H. C. & Schultz, Z. D. Digital surface enhanced Raman spectroscopy for quantifiable single molecule detection in flow. *Analyst* **149**, 3711–3715 (2024). 38895849 10.1039/d4an00801d PMC11229883

[17] Konrad, M. P., Doherty, A. P. & Bell, S. E. stable and uniform SERS signals from self-assembled two-dimensional interfacial arrays of optically coupled Ag nanoparticles. *Anal. Chem.* **85**, 6783–6789 (2013). 23751151 10.1021/ac4008607

[18] Kelly, J., Patrick, R., Patrick, S. & Bell, S. E. J. Surface-enhanced Raman spectroscopy for the detection of a metabolic product in the headspace above live bacterial cultures. *Angew. Chem. Int. Ed.* **57**, 15686–15690 (2018). 10.1002/anie.201808185 30291659

[19] Ho, C.-S. et al. Rapid identification of pathogenic bacteria using Raman spectroscopy and deep learning. *Nat. Commun.* **10**, 4927 (2019). 31666527 10.1038/s41467-019-12898-9 PMC6960993

[20] Singh, S. et al. Culture-independent Raman spectroscopic identification of bacterial pathogens from clinical samples using deep transfer learning. *Anal. Chem.* **94**, 14745–14754 (2022). 36214808 10.1021/acs.analchem.2c03391

[21] Safir, F. et al. Combining acoustic bioprinting with AI-assisted Raman spectroscopy for high-throughput identification of bacteria in blood. *Nano Lett.* **23**, 2065–2073 (2023). 36856600 10.1021/acs.nanolett.2c03015 PMC10037319

[22] Tadesse, L. F. et al. Plasmonic and electrostatic interactions enable uniformly enhanced liquid bacterial surface-enhanced Raman scattering (SERS). *Nano Lett.* **20**, 7655–7661 (2020). 32914987 10.1021/acs.nanolett.0c03189 PMC7564787

[23] Michael, I. et al. A fidget spinner for the point-of-care diagnosis of urinary tract infection. *Nat. Biomed. Eng.* **4**, 591–200 (2020). 32424198 10.1038/s41551-020-0557-2

[24] Kim, T.-H. et al. FAST: size-selective, clog-free isolation of rare cancer cells from whole blood at a liquid–liquid interface. *Anal. Chem.* **89**, 1155–1162 (2016). 27958721 10.1021/acs.analchem.6b03534

[25] Liu, L. et al. Universal method for label-free detection of pathogens and biomolecules by surface-enhanced Raman spectroscopy based on gold nanoparticles. *Anal. Chem.* **95**, 4050–4058 (2023). 36780544 10.1021/acs.analchem.2c04525

