# Supplementary material for: Nanoplasmonic SERS on fidget spinner for digital bacterial identification
Source: Microsyst Nanoeng. 2025 Mar 3;11:38. doi: 10.1038/s41378-025-00870-1 (PMC11873259; doi:10.1038/s41378-025-00870-1)
Supplement: Supplementary file 1 — Supporting Information [file 41378_2025_870_MOESM1_ESM.docx]

Supporting Information

**Nanoplasmonic SERS on Fidget Spinner for Digital Bacterial Identification**

*Mamata Karmacharya,^1^ Issac Michael,^2^ Jiyun Han,^1,2^ Elizabeth Maria Clarissa,^1,2^ Oleksandra Gulenko,^2^ Sumit Kumar,*^1,2^ Yoon-Kyoung Cho*^1,2^*

^1^Center for Algorithmic and Robotic Synthesis (CARS), Institute for Basic Science (IBS), Ulsan 44919, South Korea.

^2^Department of Biomedical Engineering, Ulsan National Institute of Science and Technology (UNIST), Ulsan 44919, South Korea.

*Correspondence to:

Sumit Kumar: sumitwithchem@gmail.com; ORCID ID: 0000-0002-4979-7677

Yoon-Kyoung Cho: [ykcho@unist.ac.kr](mailto:ykcho@unist.ac.kr); ORCID ID: 0000-0001-6423-1834

**Supporting Figures.**


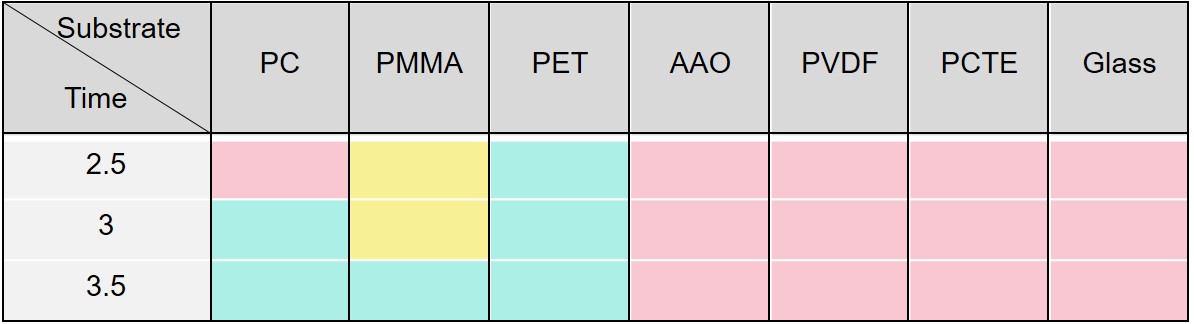


**Figure S1. Bonding of DFP with the different substrate.** Time dependent bonding of dry film photoresist (DFP) and substrate exposing at 90º C. Exposure time: 2.5, 3 and 3.5 minutes. Three different colors denote the bonding property. Turquois: bonded, Yellow: underexposed and Pink: not bonded. PC: polycarbonate, PMMA: polymethyl methacrylate, PVDF: polyvinylidene fluoride, AAO: anodic alumunium oxide, PET: polyethylene terephthalate, PCTE: polycarbonate track etch.

**
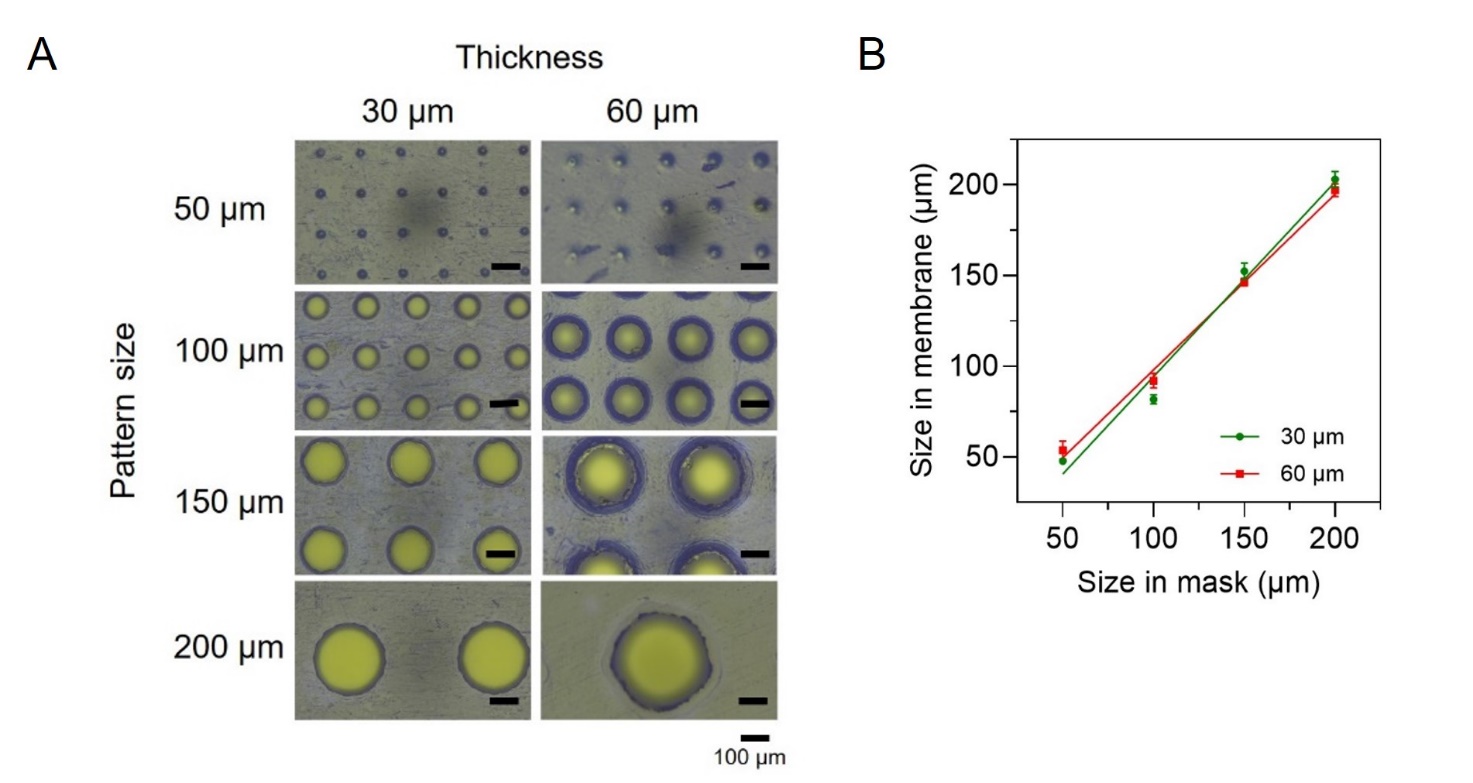
**

**Figure S2.** **Metallized feature size determination of the plasmonic-membrane.** A. Comparison of membrane pattern sizes for different membrane thicknesses. Images show metallized features (50 µm, 100 µm, 150 µm, 200 µm) in membranes with thicknesses of 30 µm (left column) and 60 µm (right column). Scale bars: 100 µm. B. Relationship between metallized feature sizes in masks and corresponding sizes in membranes for 30 µm (green circles) and 60 µm (red squares) membrane thicknesses. Data points show measured sizes with error bars representing standard deviation; n = 3 individual batches.

**
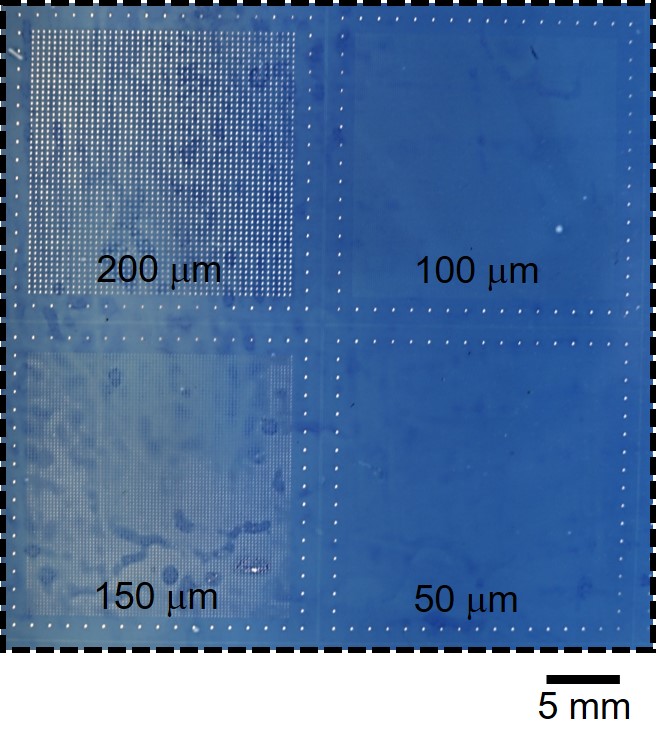
**

**Figure S3. Patterning of nitrocellulose (NC) membrane. Image showing the patterned membrane with different pattern sizes.** Pattern size on the NC membrane was generated through photolithography using the mask containing the metalized features 50, 100, 150, and 200 µm pattern. The blue color was seen due to the DFP.


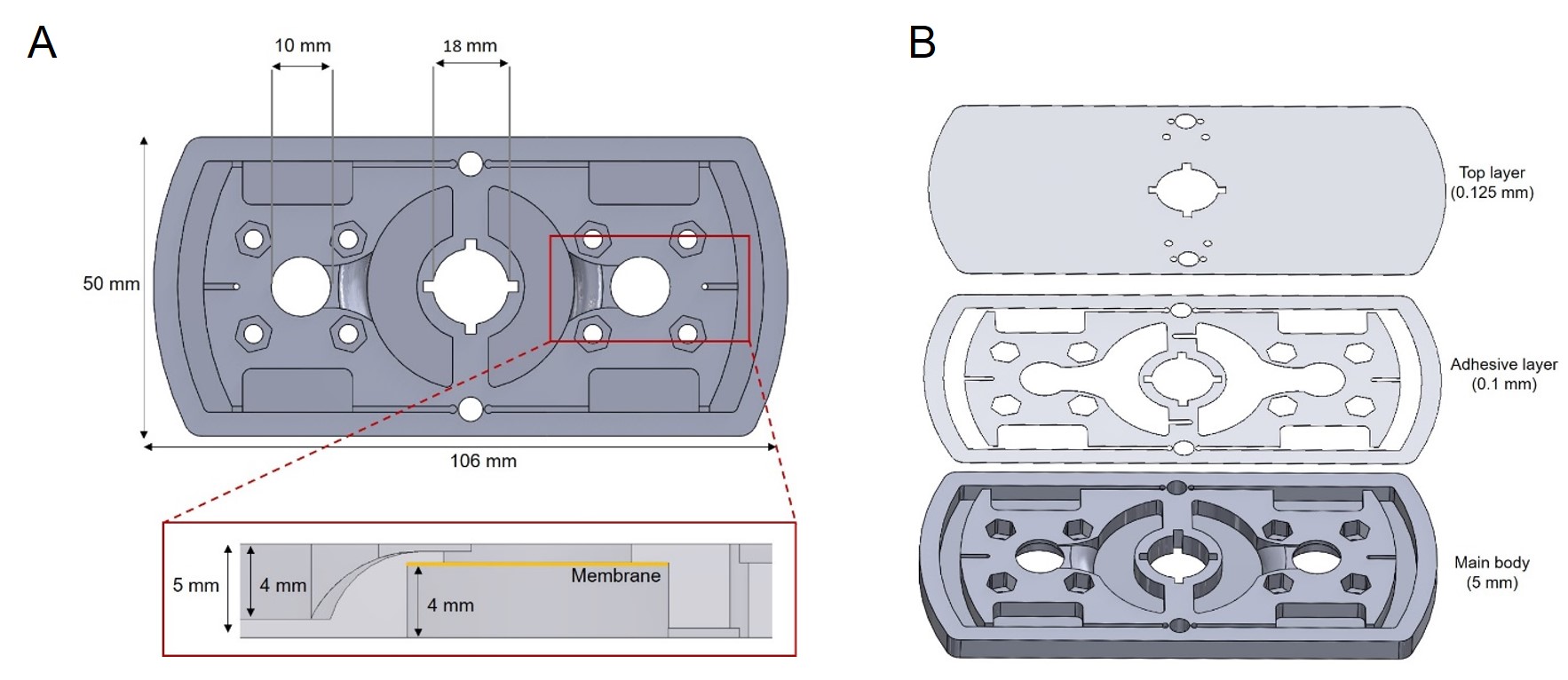


**Figure S4. Schematic diagram of the *P*-FS with detailed dimensions.** A. The top view illustrates the overall dimensions of the component, which measures 106 mm in width and 50 mm in height containing the filtration chamber of 10 mm diameter and 18 mm of bearing holder at the middle part of fidget. A red box highlights a specific area that is further detailed in the zoomed-in sectional view below. The sectional view provides a cross-sectional detail of the component, emphasizing the internal structure. The height of the section is shown to be 5 mm. Within this section, a membrane is depicted, represented by a yellow line, positioned in a 4 mm high cavity. B. Three different layers of *P*-FS: top, polycarbonate layer (0.125 mm); middle, adhesive layer (0.1 mm) and bottom layer, main body (5 mm).

**
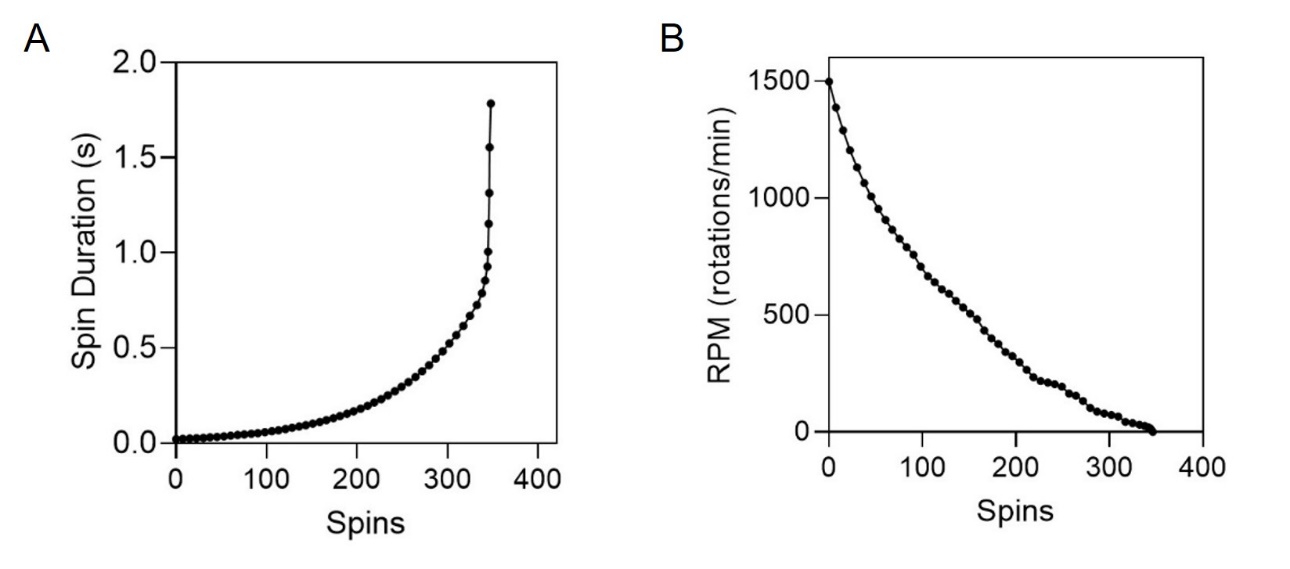
**

**Figure S5. Analysis of spinning characteristics of the *P*-FS.** The *P*-FS is actuated by a simple hand spin, similar to a conventional fidget spinner. This manual actuation generates rotational motion, which is then tracked and analyzed using the high-speed camera. A. Characterizing the speed of *P*-FS by measuring its spinning duration and rotational speed. The graph displays the spin duration (in seconds) as a function of the number of spins. The data points indicate that the spin duration increases rapidly with the number of spins, particularly after 300 spins. B. Graph presents the RPM (rotations per minute) as a function of the number of spins. The data points show a decreasing trend, with the RPM dropping sharply from around 1500 RPM at the start to nearly 0 RPM after approximately 400 spins.


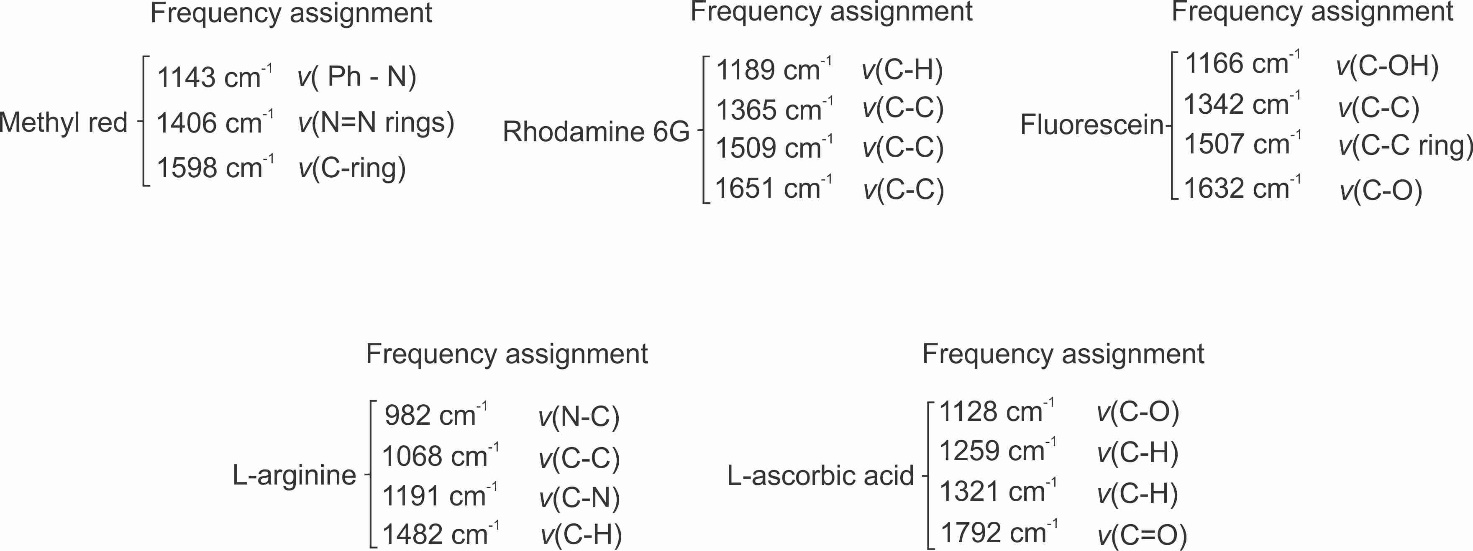


**Figure S6.** Raman shift assignments for various dye-modified polystyrene (PS) beads are listed, detailing the characteristic Raman shift frequencies (in cm⁻¹) associated with specific vibrational modes for each molecule: Methyl red, Rhodamine 6G, Fluorescein, L-arginine, and L-ascorbic acid.

**
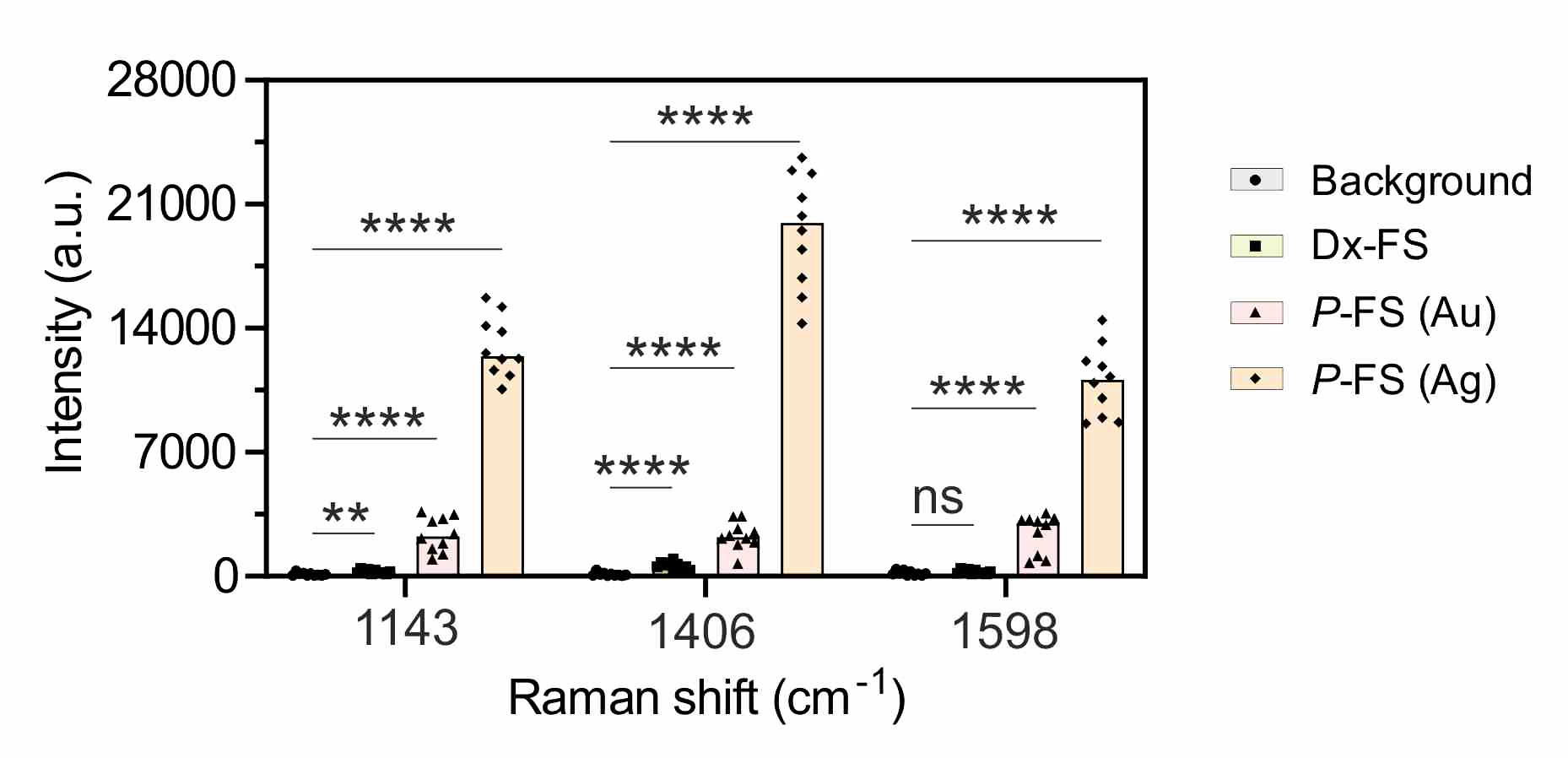
**

**Figure S7. Comparison of Raman signal intensities for different coatings on NC membrane.** The figure presents Raman spectra intensity comparisons at specific Raman shifts (1143 cm⁻¹, 1406 cm⁻¹, and 1598 cm⁻¹) for background (grey), diagnostic fidget spinner (Dx-FS, yellow), plasmonic fidget spinner with gold coating (*P*-FS (Au), pink), and plasmonic fidget spinner with silver coating (*P*-FS (Ag), orange). The intensity values are shown in arbitrary units (a.u.), and significant differences between the groups are indicated by asterisks (**p < 0.01; **p < 0.0001; ns = not significant), demonstrating the enhanced sensitivity of the *P*-FS with silver coating compared to other configurations, particularly at the 1406 cm⁻¹ peak. Data represent mean ± s.d.; n = 10 independent experiments; two-tailed unpaired Student’s t-test was used for comparing two groups.


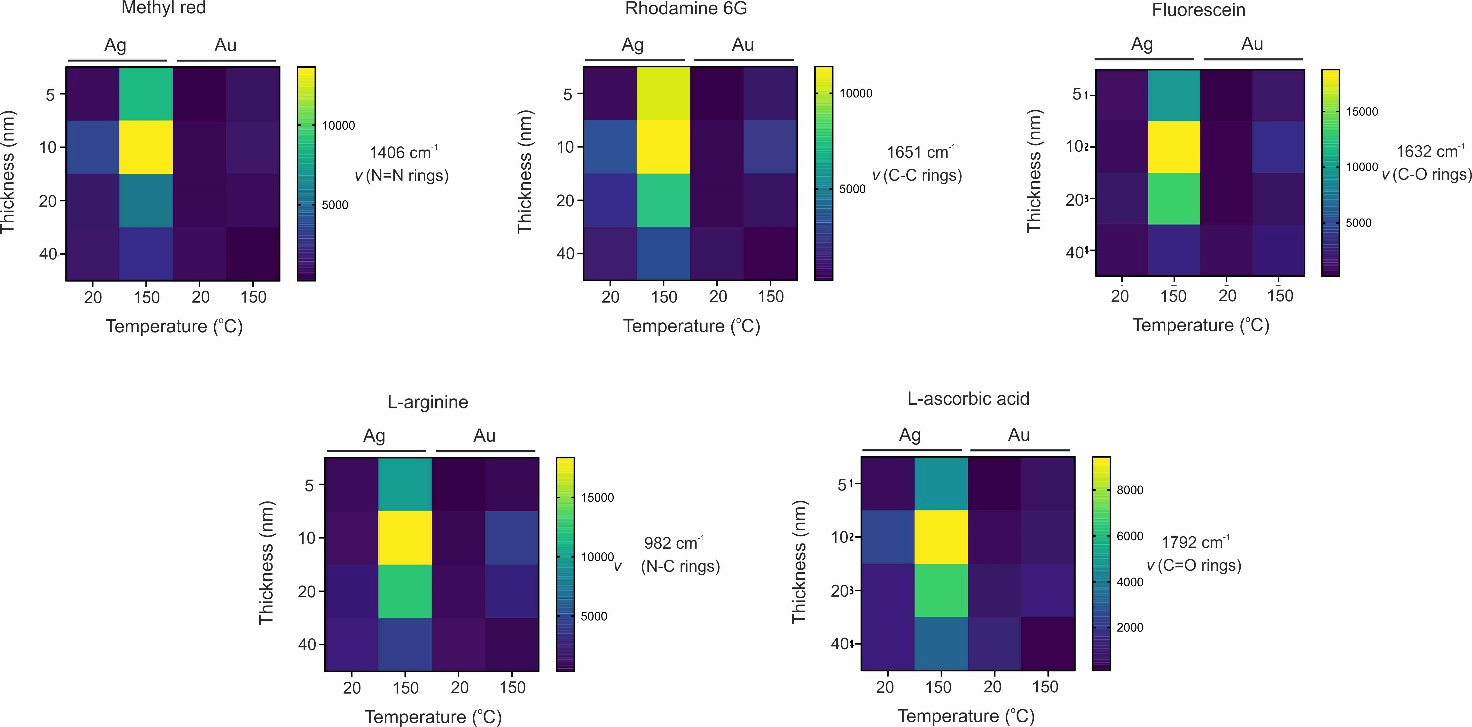


**Figure S8. Optimization of metal type, metal film thickness, and temperature on SERS intensity for various dyes on *P*-FS.** Heat maps display the SERS intensity at specific vibrational modes for different dye-coated PS: methyl red (1406 cm⁻¹, ν(N=N rings)), rhodamine 6G (1651 cm⁻¹, ν(C-C rings)), fluorescein (1632 cm⁻¹, ν(C-O rings)), L-arginine (982 cm⁻¹, ν(N-C rings)), and L-ascorbic acid (1792 cm⁻¹, ν(C=O rings)). The SERS intensities were measured across varying metal film thicknesses (5 nm, 10 nm, 20 nm, and 40 nm) and temperatures (20°C and 150°C) for silver (Ag) and gold (Au) films. The color scale represents SERS intensity, with yellow indicating higher intensities and blue/purple indicating lower intensities. The results demonstrate the influence of film thickness and temperature on SERS performance, with significant differences observed between Ag and Au films, highlighting the importance of optimizing these parameters for effective SERS-based detection.

**
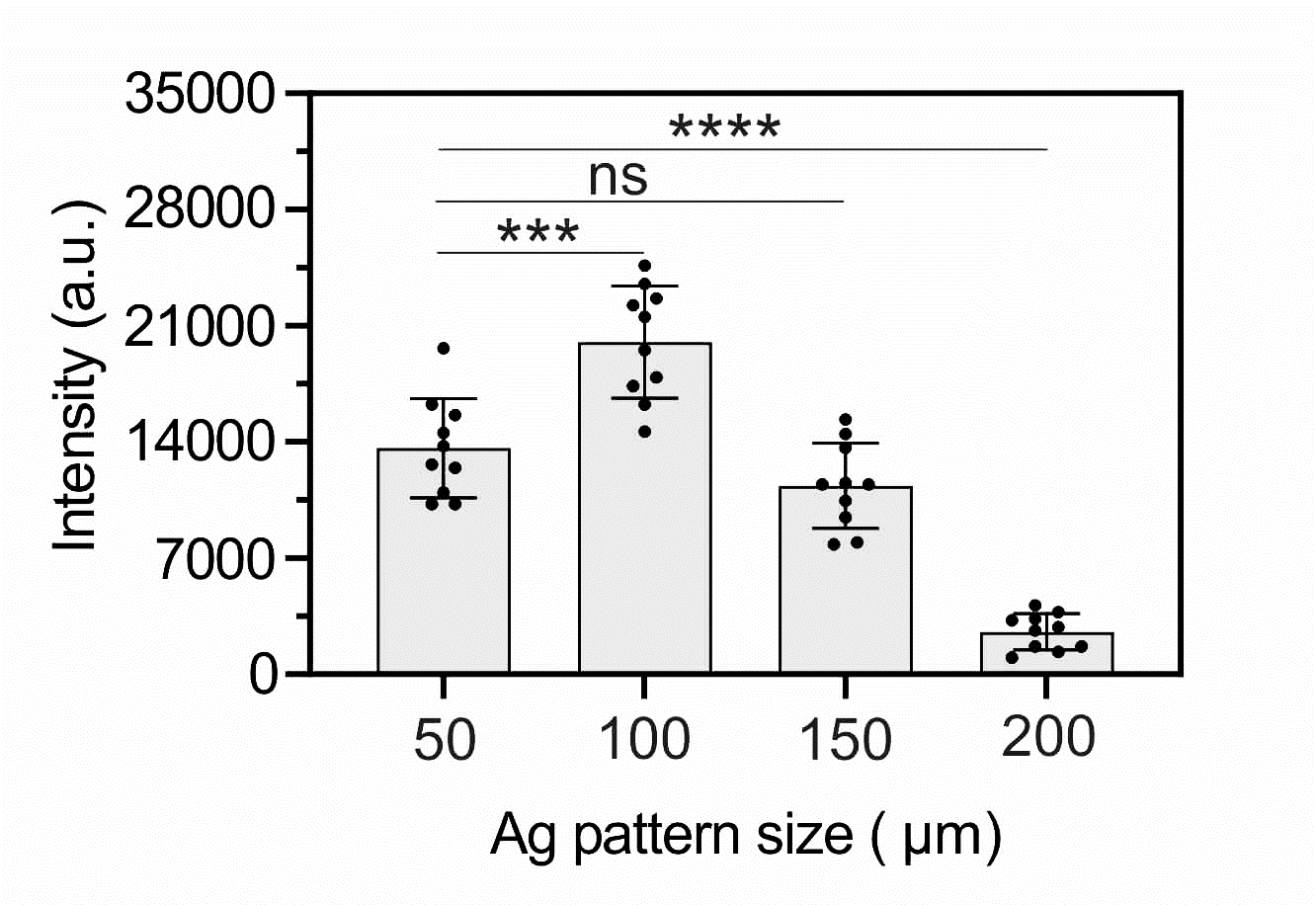
**

**Figure S9. SERS intensity as a function of Ag pattern size on plasmonic membranes.** The bar graph illustrates the SERS intensity (in arbitrary units, a.u.) corresponding to different silver pattern sizes (50 µm, 100 µm, 150 µm, and 200 µm) on plasmonic membranes of *P*-FS. The data points indicate individual measurements, while the error bars represent the variability within the data. The pattern size of 100 µm produced the highest SERS intensity, suggesting optimal hotspot generation for enhanced Raman signal detection at this pattern size. Statistical significance is denoted by asterisks (**p < 0.001; **p < 0.0001; ns = not significant), indicating significant differences in SERS intensity between certain pattern sizes, particularly between 100 µm and other sizes. Data represent mean ± s.d.; n = 10 independent fusion batches; two-tailed unpaired Student’s t-test was used for comparing two groups.

**
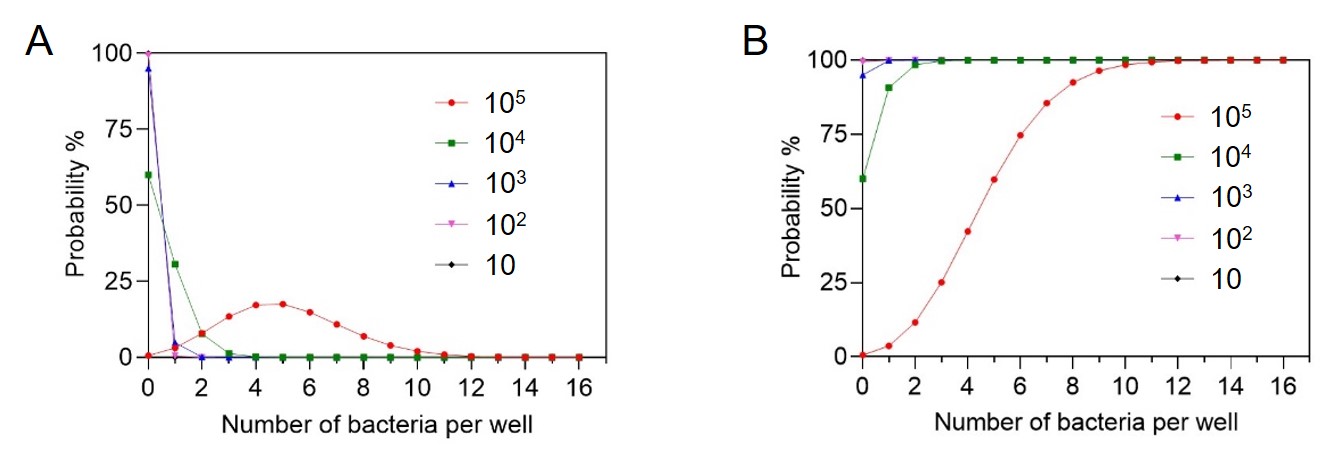
**

**Figure S10.** **Distribution of bacteria on the metalized features of the membrane.** A. Probability of a metalized features containing κ bacteria, given the average bacteria concentration λ per metalized features using the Poisson distribution, p(κ, λ) = λ^κ^e^-λ^/κ!. B. The Poisson cumulative distribution indicates that over 90% of metalized features contain between 1 and 9 bacteria at concentrations ranging from 10 to 10^5^ bacteria per mL. These concentrations correspond to λ values of 5.10, 0.51, 0.051, 0.0051, and 0.00051, respectively.

**
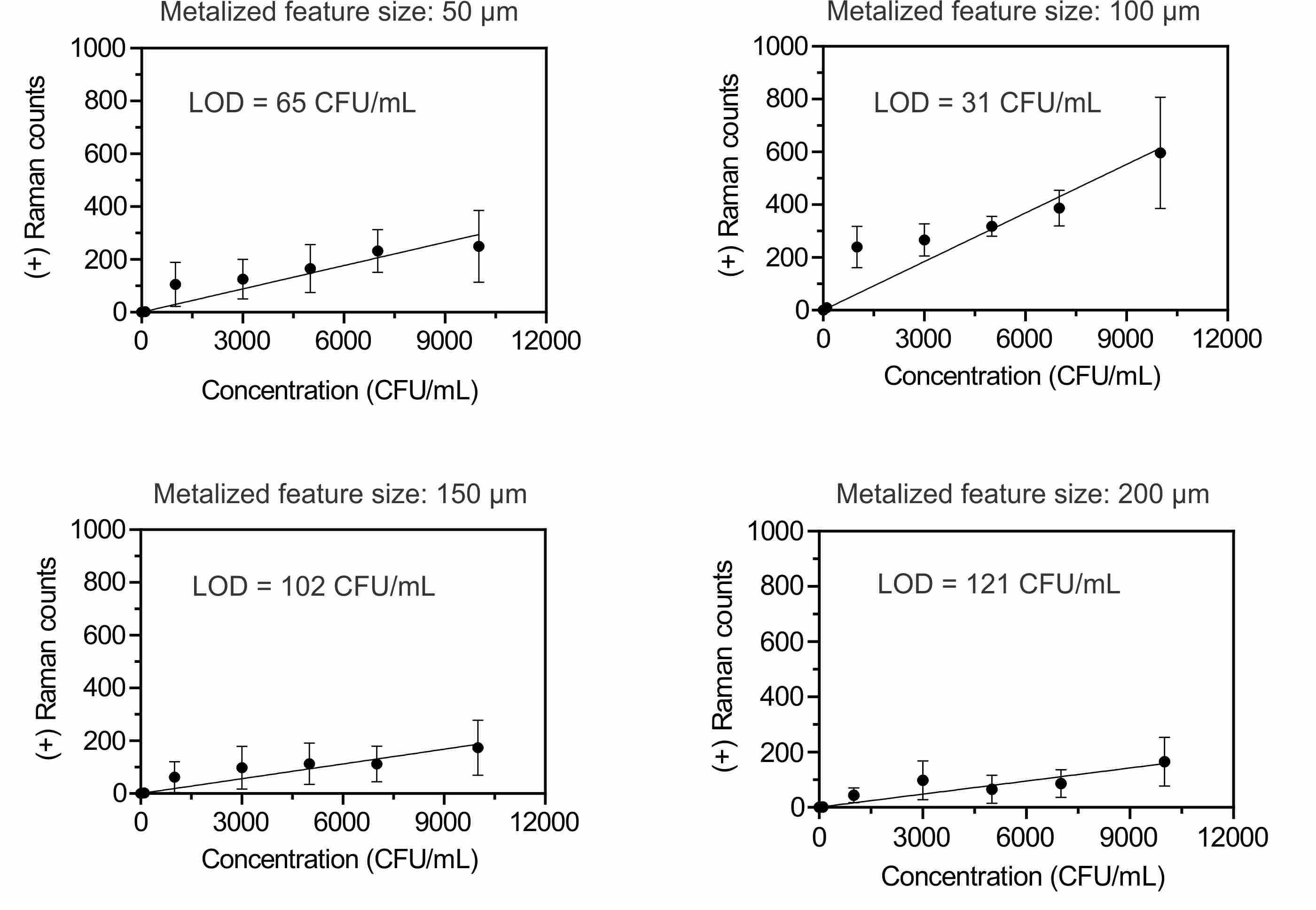
**

**Figure S11. Limit of detection (LOD) comparison for different pattern sizes on plasmonic membranes.** Graphs show the relationship between bacterial concentration (CFU/mL) and Raman counts for plasmonic membranes with metalized features sizes of (a) 50 µm, (b) 100 µm, (c) 150 µm, and (d) 200 µm. The LOD values are indicated for each pattern size, demonstrating that the 100 µm metalized feature achieves the lowest LOD at 31 CFU/mL, highlighting its superior sensitivity for bacterial detection. Data represent mean ± s.d.; n = 3 digital counting batches.

**
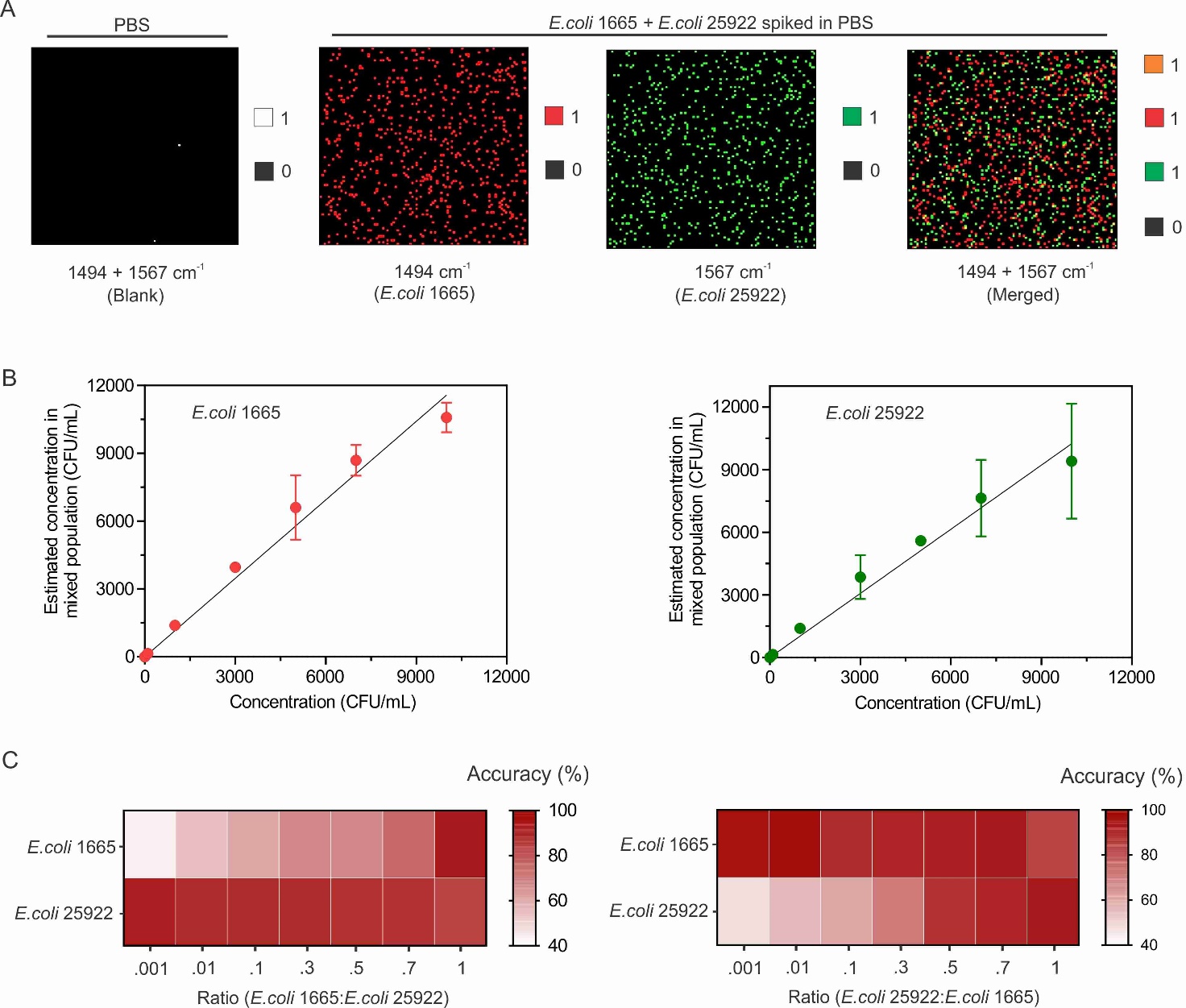
**

**Figure S12.** **Detection and quantification of *E. coli* 1655 and *E. coli* 25922 in mixed populations using SERS-based *P*-FS.** (A) SERS intensity maps showing the detection of *E. coli* 1655 and *E. coli* 25922 in phosphate-buffered saline (PBS). The blank sample (leftmost image) serves as a negative control with no detectable Raman signals. The second and third panels represent individual detection of *E. coli* 1655 (1494 cm⁻¹, red) and *E. coli* 25922 (1567 cm⁻¹, green), respectively, while the rightmost panel shows the merged detection of both strains in a mixed population. The binary "1" and "0" assignments indicate the presence or absence of Raman signals for each species. (B) Calibration curves for *E. coli* MG1655 (left) and *E. coli* 25922 (right) showing the estimated concentration in mixed populations versus actual concentration (CFU/mL). The linear relationship demonstrates the effectiveness of the digital counting method for quantifying bacterial concentrations. Data represent mean ± SD; n = 3 independent experiments. (C) Accuracy heatmaps showing the percentage accuracy in detecting and quantifying *E. coli* 1655 and *E. coli* 25922 at various concentrations in mixed populations. Higher concentrations result in greater accuracy, while accuracy decreases at lower concentrations due to signal overlap and reduced intensity. The color scale represents the detection accuracy (%) for each concentration level.

**Table S1. Media and incubation conditions used for the different bacteria.**

| **Bacterial species** | **Isolate code** | **Solid Media** | **Liquid Media** | **Temp (°C)** | **Incubation Atmosphere** | **Speed (rpm)** |
| --- | --- | --- | --- | --- | --- | --- |
| *E. coli* | 25922 | LB agar | LB broth | 37 | Air | 150 |
| *E. coli* | MG1655 | LB agar + 100 µg Ampicillin | LB agar + 100 µg Ampicillin | 37 | Air | 150 |
| *S. aureus* | 25923 | LB agar | LB agar | 37 | Air | 150 |
| *S. mutan* | 3065 | BHI agar + 1% sucrose | BHI agar + 1% sucrose | 37 | 5% CO_2_ | 0 |
| *L. brevis* |  | Lactobacilli MRS agar | Lactobacilli MRS agar | 30 | Air | 0 |

LB: Lueria-Bertenni, BHI: Brain Heart Infusion and MRS: deMan, Rogosa and Sharpe
